# Gene X environment: the cellular environment governs the transcriptional response to environmental chemicals

**DOI:** 10.1186/s40246-020-00269-1

**Published:** 2020-05-24

**Authors:** Andreanna Burman, Rolando Garcia-Milian, Shannon Whirledge

**Affiliations:** 1 Department of Obstetrics, Gynecology, and Reproductive Sciences, Yale School of Medicine, 310 Cedar St, PO Box 208063, New Haven, CT 06520 USA; 2 Bioinformatics Support Program, Cushing/Whitney Medical Library, Yale School of Medicine, New Haven, CT 06520 USA

**Keywords:** Gene expression array, Gene ontology analysis, Environmental chemical, Gene-environment interaction, Xenoestrogen

## Abstract

**Background:**

An individual’s response to environmental exposures varies depending on their genotype, which has been termed the gene-environment interaction. The phenotype of cell exposed can also be a key determinant in the response to physiological cues, indicating that a cell-gene-environment interaction may exist. We investigated whether the cellular environment could alter the transcriptional response to environmental chemicals. Publicly available gene expression array data permitted a targeted comparison of the transcriptional response to a unique subclass of environmental chemicals that alter the activity of the estrogen receptor, xenoestrogens.

**Results:**

Thirty xenoestrogens were included in the analysis, for which 426 human gene expression studies were identified. Comparisons were made for studies that met the predefined criteria for exposure length, concentration, and experimental replicates. The cellular response to the phytoestrogen genistein resulted in remarkably unique transcriptional profiles in breast, liver, and uterine cell-types. Analysis of gene regulatory networks and molecular pathways revealed that the cellular context mediated the activation or repression of functions important to cellular organization and survival, including opposing effects by genistein in breast vs. liver and uterine cell-types. When controlling for cell-type, xenoestrogens regulate unique gene networks and biological functions, despite belonging to the same class of environmental chemicals. Interestingly, the genetic sex of the cell-type also strongly influenced the transcriptional response to xenoestrogens in the liver, with only 22% of the genes significantly regulated by genistein common between male and female cells.

**Conclusions:**

Our results demonstrate that the transcriptional response to environmental chemicals depends on a variety of factors, including the cellular context, the genetic sex of a cell, and the individual chemical. These findings highlight the importance of evaluating the impact of exposure across cell-types, as the effect is responsive to the cellular environment. These comparative genetic results support the concept of a cell-gene-environment interaction.

## Background

Disease risk or variation in disease susceptibility across populations reflects the complex interaction between an individual’s genotype and their environment [1]. This model of gene-environment interactions holds the potential for targeted interventions to high-risk groups for broad public health benefit [2]. However, few studies have identified candidate genes or their genetic alterations that possess a significant interaction with the environment and disease risk [3–5]. One potential challenge to detecting gene-environment interactions is the assumption that genes are regulated and expressed in consistent manner across all cell-types. Instead, the transcription of DNA sequences into biologically relevant mRNA is mediated by the context of that cell, such that transcript levels of specific genes vary across cell-types [6, 7]. While specificity in gene regulation allows specialized cells to perform distinct functions essential to the biology of the resident tissue, this specificity argues that the cellular environment may also modulate disease risk when considering gene-environment interactions.

Transcription factor activity is gated by a number of factors, including DNA sequence, chromatin environment, the presence or absence of regulatory co-factors, post-translational modifications to the transcription factor, and ligand chemistry. For example, the genes regulated by 17β-estradiol (E_2_)-activated estrogen receptor alpha (ERα) were found to be specific to cervical or kidney cells, dependent on the co-factors present [8]. In addition, minor changes in the chemical structure of the endogenous estrogens (E_1_, E_2_, and E_3_) can alter the activity of ligand-bound ER. ERα can bind both estradiol (E_2_) and the metabolite of estradiol, estrone (E_1_), but when comparing the transcriptional response to these closely related ligands, the type and magnitude differs [9–11]. Man-made chemicals that closely resemble the structure of endogenous ligands can further diversify the molecular response of a transcription factor.

Endocrine disrupting chemicals (EDCs) are exogenous compounds that can mimic or block the activity of endogenous hormones through interactions with their receptors, thereby affecting the normal function of these hormones. EDCs are encountered through a variety of mechanisms, including pharmaceuticals, food additives, plastic bottles, detergents, herbicides/insecticides, flame retardants, personal care products, toys, and consumer food packaging. Xenoestrogens are a well-characterized subclass of EDCs that mimics some structural characteristics of the endogenous estrogen compounds, and therefore, can act as estrogens or interfere with the actions of endogenous estrogens on the ER. Estrogen signaling plays an important role in the physiology of many organs, including the brain, vascular system, skeletal muscle, bone, adipose tissue, mammary gland, ovary, and uterus [12–15]. As such, chemicals that alter the activity of endogenous estrogens or inappropriately activate the estrogen signaling pathway can disturb the normal physiology of these organs, contributing to disease risk.

Due to the environmental abundance and the physiological implications of xenoestrogen exposure, numerous studies have measured the transcriptional response to xenoestrogens in a variety of human cell-types, although the individual studies largely focused on one cell-type [16–19]. Yet, the cellular plasticity in transcription factor activity suggests that the outcome of xenoestrogen exposure in one cell-type may not be universal. Functional assays have demonstrated that EDCs produce unique biological responses depending on the cell-type assayed. For example, 2,3,7,8-tetrachlorodibenzo-p-dioxin (TCDD) inhibits proliferation in colorectal and liver cancer cells but increases proliferation in human keratinocytes [20–22].

In this study, we evaluate publicly available gene expression data using an unbiased approach to determine the transcriptional response to specific xenoestrogen across cell-types and to compare the effects of various xenoestrogens when controlling for cell-type. We hypothesized that the cellular environment, including organ of origin and genetic sex, would be important factors mediating the transcriptional response to environmental chemicals. To assess the difference in gene expression induced by a single xenoestrogen across different cell-types, datasets were collected from human breast, liver, and endometrial cells treated with genistein. We then evaluated the transcriptional effects of several EDCs (TCDD, bisphenol A (BPA), genistein, diethylstilbestrol (DES), and ethinyl estradiol (EE2)) in a single human liver cell line. Sex-related differences were evaluated by comparing the gene expression profiles of human liver HepG2 and HepaRG cells, which originated from a male and female subject, respectively. Our findings show that the cellular environment is an important determining factor when evaluating the response to environmental chemicals. Thus, human public health genomics and toxicogenomics studies should consider multiple cellular sources when predicting EDC risk.

## Methods

### Array dataset collection

The National Center for Biotechnology Information (NCBI) Gene Expression Omnibus (GEO) DataSets (https://www.ncbi.nlm.nih.gov/gds) and the EMBL-European Bioinformatics Institute ArrayExpress (https://www.ebi.ac.uk/arrayexpress/) databases were utilized to identify publicly available gene expression data. Databases were initially queried in November 2018 and reevaluated for updates in October 2019. Searches within GEO were filtered by “Expression profiling by array” and “Expression profiling by high throughput sequencing.” Searches within ArrayExpress were filtered by RNA assays, specifically array and sequencing assays. Results were limited by organism to *Homo sapiens*. GEO and ArrayExpress were queried for datasets from human cell lines treated with the following chemicals: Genistein (GEN), Bisphenol A (BPA), 2,3,7,8-Tetrachlorodibenzo-p-dioxin (TCDD), Polychlorinated bisphenol (77, 153, 138, 126; PCB), 17α-Ethinylestradiol (EE2), 4-Nonylphenol (NP), Di-2-ethylhexyl phthalate (DEHP), Estrone, Daidzein, Diethylstilbestrol (DES), Dichlorodiphenyltrichloroethane (DDT), Methoxychlor (MOC), Atrazine, Bisphenol S (BPS), Bisphenol AF (BPAF), Benzophenone-2, Zearalenone, Bisphenol B (BPB), Testosterone propionate, Triphenylethylene, 3-Tetramethylbutyl, 4-Cumyphenol, 4-Dodecylphenol, 5HPP-33, Dodecylphenol, Equilin, Ethylhexylparaben, Meso-hexestrol, Mestranol, and Norgestrel. The structures for these chemicals were created using ChemDraw 18.0 (PerkinElmer, Waltham, MS, USA). The following information was collected from each dataset: GEO Series (GSE) accession number, cell line, genotypic sex of the cell, EDC name, treatment concentration, incubation time, number of replicates, and GEO Sample (GSM) numbers of both the chemical-treated samples and intra-experimental controls (Supplemental Table 1).

### Identification of differentially expressed genes

The raw data files were imported to the Partek Genomics Suite 6.6 software as CEL files (Partek, St. Louis, MO, USA). Datasets were analyzed using the “Gene Expression” workflow. Imported samples were assigned a categorical attribute for treatment. The principal component analysis was visualized by attribute (Supplemental Figure 1). Differentially expressed genes were detected by selecting the attribute as the interaction for the ANOVA and contrasting treatment vs. control. Lists of differentially regulated genes were created by using the contrast function with the settings of “have any change” and “*p* value < 0.05.” The gene symbol and title, *p* value, and fold change data were exported for further analyses. InteractiVenn (http://www.interactivenn.net/) created Venn diagrams to visualize the unique and commonly regulated genes within the lists of differentially expressed genes [23].

### Gene ontology analysis

Differentially expressed genes that met statistical significance were analyzed with the Ingenuity Pathway Analysis software (IPA; Qiagen, Valencia, CA, USA) to determine gene annotations. Gene set enrichment for the canonical signaling pathways and molecular and cellular functions was determined by IPA using the Fisher’s exact test with a cutoff of *p* < 0.05. Pathways and functions were ranked using the ratio of the number of genes from the dataset that mapped to the pathway divided by the total number of genes mapped in that pathway.

## Results

### Identification of available gene expression datasets and inclusion criteria

To determine whether chemicals with known ER activity would demonstrate transcriptional plasticity in response to the cellular environment, we searched the NCBI GEO and ArrayExpress databases for gene expression data in which various human cell lines were treated with xenoestrogens [24]. We identified 91 publicly available gene expression profiling series in the GEO and ArrayExpress databases, which included 426 unique datasets for the chemicals searched (Supplemental Table 1). We found gene expression data for 18 of the 30 queried xenoestrogens: GEN, BPA, TCDD, PCBs, EE2, DEHP, NP, DDT, Daidzein, DES, Estrone, MOC, BPS, Atrazine, BPAF, Benzophenone-2, BPB, and Zearalenone (Supplemental Table 2). GEN and BPA had the greatest number of datasets available (97 and 94, respectively), while 3 xenoestrogens, Benzophenone-2, BPB, and Zearalenone only had 1 dataset available (Supplemental Table 3). Overall, the immortalized breast cancer cell line MCF-7 (ATCC® HTB-22™) was the most represented human cell-type, with 155 gene expression datasets, followed by HepG2 (ATCC® HB-8065™) (*n* = 51) and Ishikawa (ATCC® 13,347™) (*n* = 48).

**Fig. 1:**
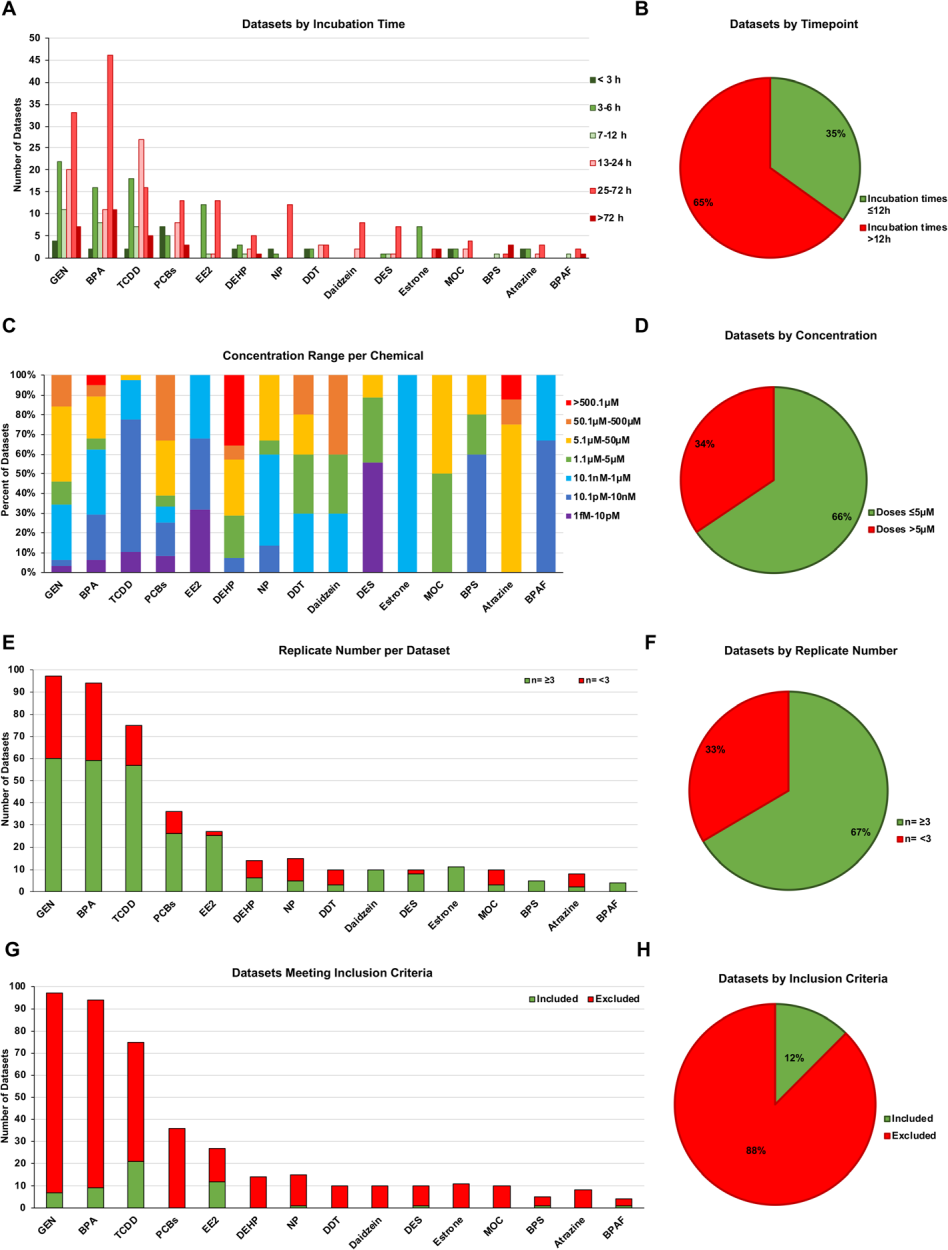
Xenoestrogen datasets evaluated by exposure length, concentration, and number of experimental replicates. **a** Datasets sorted by the incubation time of chemical exposure and graphed by xenoestrogen. **b** Datasets grouped by incubation times ≤ 12 h (green) and > 12 h (red). Criteria for subsequent analysis ≤ 12 h. **c** The concentration ranges of chemicals per dataset. **d** Datasets grouped by concentrations ≤ 5 μM (green) and > 5 μM (red). Criteria for subsequent analysis ≤ 5 μM. **e** Datasets sorted by the number of experimental replicates where *n* ≥ 3 is depicted in green and *n* < 3 is red. **d** Datasets grouped by replicate number. Criteria for subsequent analysis *n* ≥ 3. **f** Datasets congruous with analysis criteria (incubation time, concentration range, and replicate number) were graphed by chemical (green). Datasets not meeting all three criteria are depicted in red. **g** Datasets grouped by inclusion (green) and exclusion (red) from subsequent analysis

In order to understand the direct transcriptional response of each xenoestrogen, datasets were sorted by incubation time (Fig. 1a). For most of the datasets, cells were treated for relatively long incubation periods (> 24 h), which may reflect secondary or tertiary effects of the xenoestrogen on transcription. To focus on primary effects, we applied a timepoint cutoff of ≤ 12 h for the inclusion of gene expression datasets. Interestingly, only 35% of the datasets were studies with an experimental endpoint 12 h or less (Fig. 1b). The concentration of the chemical treatment is also an important factor for translating in vitro studies to relevant, real-world exposures. Therefore, the identified datasets were grouped by treatment concentration (Fig. 1c). The gene expression datasets included experimental doses ranging from 3 fM to 200 mM, and the concentration range varied greatly by chemical. For example, we identified datasets for BPA ranging from 1 pM to 200 mM. Moreover, all experiments performed with EE2, Estrone, and BPAF utilized a treatment exposure of less than 5 μM, while all gene expression datasets from atrazine-treated cells utilized an exposure of > 5.1 μM. To more closely relate to potential environmental exposures, we selected a treatment exposure of ≤ 5 μM as the cutoff for gene expression data to be included in our comparative analyses. Therefore, all atrazine datasets were excluded from subsequent analyses based on the exposure dose, and 34% of the identified datasets did not meet the inclusion criteria (Fig. 1d). Of note, to make the comparison across xenoestrogens to the endogenous estrogen estradiol (E_2_) in one cell-type, the lowest concentration of E_2_ applied to HepG2 and HepaRG cells was 30 μM (Supplemental Table 4) [25]. Finally, gene expression datasets were evaluated by replicate number for each treatment group (Fig. 1e). To perform statistical analysis, it was necessary that each treatment group have at least 3 experimental replicates. We found that 33% of the gene expression datasets did not have a sufficient number of replicates (Fig. 1f). The overall inclusion criteria for analysis was ≤ 12 h exposure time, ≤ 5 μM dose, and ≥ 3 experimental replicates (Fig. 1g). When these criteria were applied collectively, only 12% of the 426 identified gene expression datasets met the inclusion criteria (Fig. 1h). Furthermore, none of the studies where cells were treated with PCB’s, DEHP, DDT, Daidzein, Estrone, MOC, or Atrazine met the inclusion criteria, and these chemicals are not represented in the subsequent analyses. The examination of publicly available datasets demonstrates that experimental design with environmental chemicals is highly variable, restricting the ability for effects to be compared across datasets.

### The cellular environment mediates the transcriptional response to genistein

**Fig. 2:**
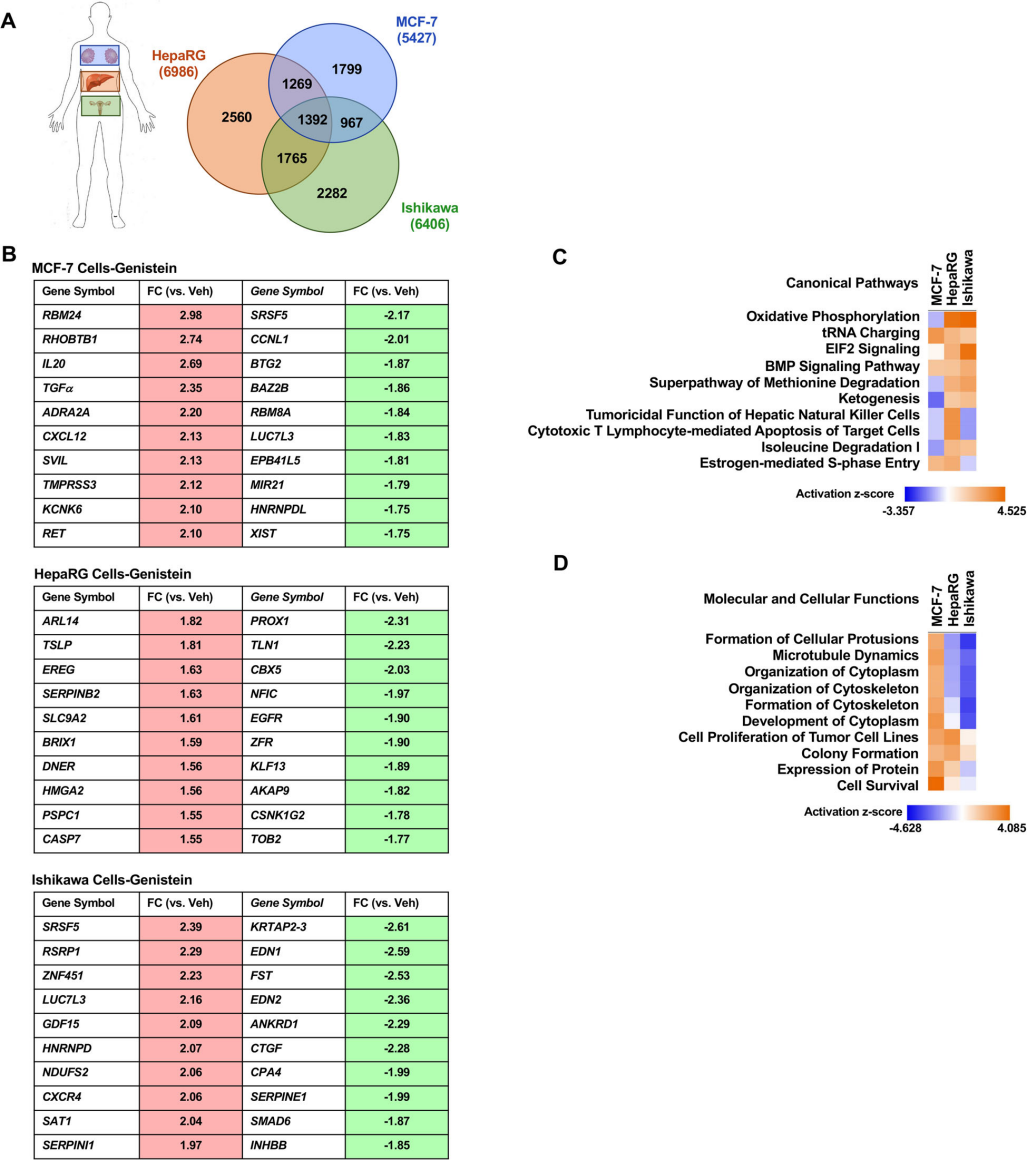
Transcriptional response to genistein across cell-types. Gene expression data was identified for cells treated with 1 μM genistein for 6 h. **a** The genistein-regulated genes within each cell line were compared by a Venn diagram to identify unique and commonly regulated genes. **b** The top 10 induced and repressed genes are listed by cell line. **c** A comparison analysis of the most significantly associated canonical pathways sorted by predicted activation *z* score. **d** A comparison analysis of the top molecular and cellular functions sorted by predicted activation *z* score

Controlling for chemical and genetic sex, we identified three cell-types to evaluate whether the cellular environment influences the transcriptional response to xenoestrogens, MCF-7 (breast), HepaRG (liver), and Ishikawa (endometrium). The datasets were derived from experiments in which all cell lines were treated for 6 h with 1 μM GEN, and these datasets were previously reported as part of a larger connectivity map of gene arrays from multiple chemicals [26]. A Venn diagram was created to visualize the unique and commonly regulated genes (Fig. 2a). GEN exposure resulted in the cell-specific regulation of several thousand genes per cell-type: 1799 unique genes in MCF-7 cells, 2560 unique genes in HepaRG cells, and 2282 unique genes in Ishikawa cells. Despite an identical experimental design, less than 25% of the significantly regulated genes in each cell-type were shared with the other two cell-types. We determined the top 10 induced and repressed genes by cell-type (Fig. 2b). Remarkably, none of the top regulated genes were shared by the three cell-types. LUC7 like 3 pre-mRNA splicing factor (*LUC7L3*) and serine- and arginine-rich splicing factor 5 (*SRSF5*) were common to two cell-types but repressed in MCF-7 and induced in Ishikawa cells. These data show that a cellular environment strongly influences the transcriptional response to environmental chemicals.

To understand the signaling pathways and biological functions related to those transcriptional changes induced by GEN exposure, we examined gene annotations using the IPA gene ontology software. A gene enrichment comparison analysis of the canonical signaling pathways, including an assigned activation z-score that infers the likely activation status of each pathway, was performed for the genes regulated by GEN in the three cell lines (Fig. 2c; comparison based on *p* value in Supplemental Figure 2). The predicted activation *z* score varied by cell-type, with only “tRNA Charging” and “BMP Signaling Pathway” having a similar activation status across cell-types. The heatmap of the top Molecular and Cellular Functions also displayed variability in the activation *z* score by cell-type (Fig. 2d). Here, the functions regulated by GEN were more similar in HepaRG and Ishikawa cells compared to MCF-7 cells. Interestingly, functions related to cytoplasmic or cytoskeletal organization were altered in an opposing manner by GEN in MCF-7 cells compared to HepaRG and Ishikawa cells. These findings demonstrate that the biological effect of environmental chemicals is mediated by the cell-type exposed, demonstrating an interaction between the gene, cellular environment, and biological response.

### Xenoestrogens induce a unique transcriptional response in HepG2 cells

**Fig. 3:**
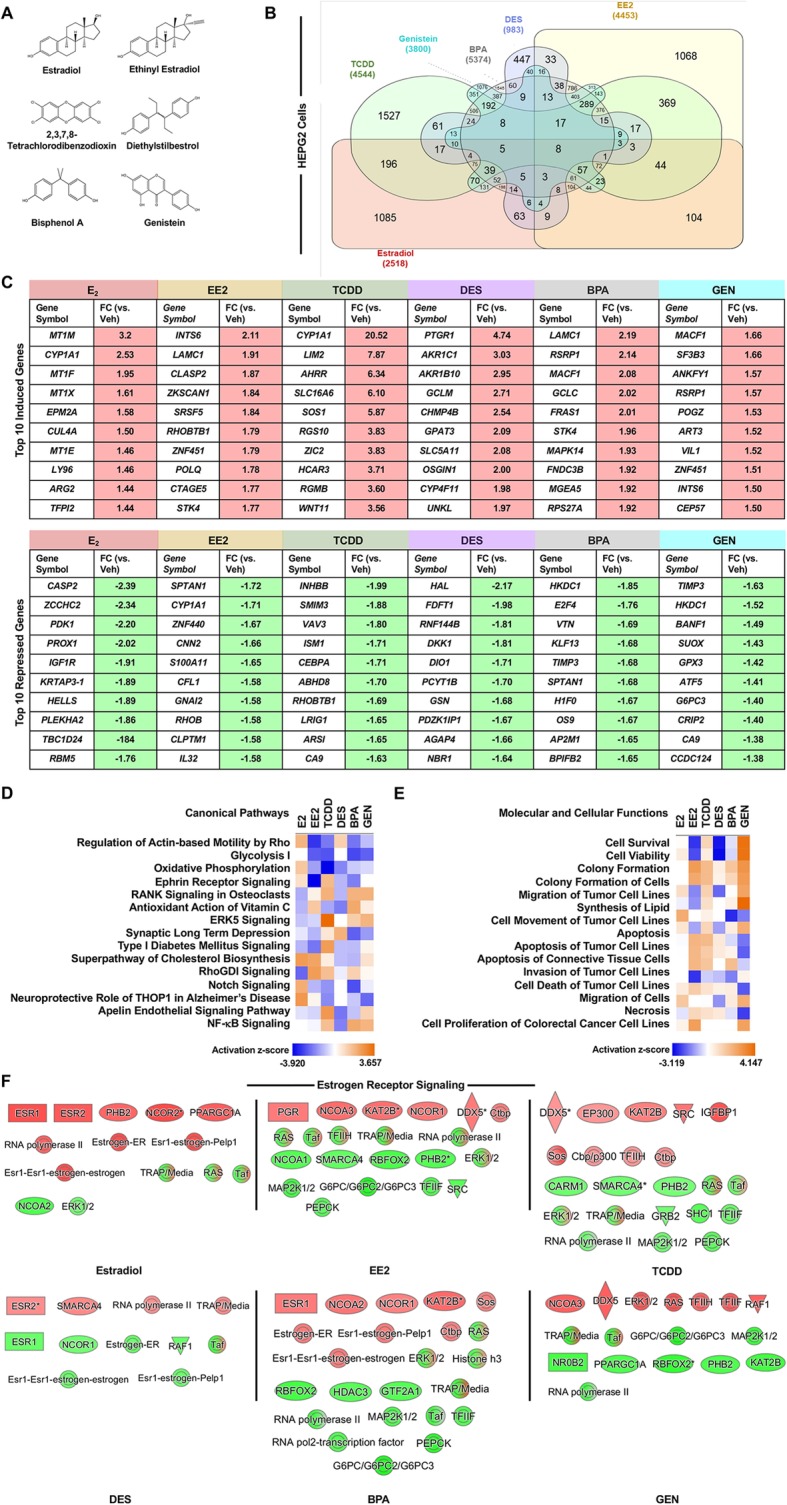
Comparison of transcriptional response controlling for cell-type. Differentially expressed genes were identified for HepG2 cells exposed to 1 μM ethinyl estradiol (EE2), 10 nM 2,3,7,8-tetrachlorodibenzodioxin (TCDD), 5 μM diethylstilbestrol (DES), 1 μM bisphenol A (BPA), or 1 μM genistein (GEN) for 6 h or 30 μM estradiol (E_2_) for 12 h. **a** The chemical structures of E_2_, EE2, TCDD, DES, BPA, and GEN were created using Chemdraw 18.0. **b** The xenoestrogen-regulated genes in HepG2 cells were compared by a Venn diagram to identify unique and commonly regulated genes. **c** The top 10 induced and repressed genes are listed by chemical. **d** A comparison analysis of the top canonical pathways utilizing the predicted activation *z* score. **e** A comparison analysis of the top molecular and cellular functions utilizing the predicted activation *z* score. **f** Induced (red) and repressed (green) molecules associated with the “Estrogen Receptor Signaling” pathway visualized for each chemical

Although many of the xenoestrogens share a similar chemical structure and binding affinity for estrogen receptors, the receptor conformation induced by xenoestrogen binding and the biological response is unique (Fig. 3a) [27–29]. Therefore, we hypothesized that each xenoestrogen would produce a distinct transcriptional response. To compare the transcriptional response produced in response to various xenoestrogens while controlling for cell-type, differentially expressed genes from datasets in HepG2 cells meeting the inclusion criteria were compared. Datasets were available for BPA, DES, EE2, GEN, and TCDD, which were included in previously published studies [26, 30]. In these datasets, HepG2 cells were treated for 6 h (1 μM BPA, 1 μM EE2, 1 μM GEN, or 10 nM TCDD) or 12 h (5 μM DES). Genes differentially regulated by xenoestrogens were compared to those differentially regulated by E_2_ in HepG2 cells.

A Venn diagram was constructed to visualize the overlapping and uniquely regulated genes (Fig. 3b). BPA treatment resulted in the greatest number of differentially regulated genes (5374), while DES treatment generated the least (983). We identified eight genes that were commonly regulated by E_2_ and the five xenoestrogens: rho guanine nucleotide exchange factor 12 (*ARHGEF12*), tight junction protein 2 (*TJP2*), sorbin and SH3 domain containing 2 (*SORBS2*), solute carrier family 25 member 37 (*SLC25A37*), eukaryotic translation initiation factor 3 subunit L (*EIF3L*), AF4/FMR2 family member 4 (*AFF4*), death associated protein 3 (*DAP3*), and tumor protein P73 (*TP73*). When comparing the differentially expressed genes of the five xenoestrogens evaluated, BPA treatment shared the most genes in common with E_2_ (706), while DES treatment shared the least in common with E_2_ (163). Moreover, each xenoestrogen produced a subset of uniquely regulated genes, not shared with E_2_ or the other xenoestrogens (Supplemental Table 5). We determined the top 10 genes induced by treatment and the top 10 genes repressed by treatment (Fig. 3c). Although no gene was common to all treatments, some genes were shared between several groups. For example, cytochrome P450 family 1 subfamily A member 1 (*CYP1A1*) was induced by E_2_ and TCDD and repressed by EE2.

To determine the functional significance of the differentially regulated genes, we evaluated gene annotations using the IPA gene ontology software. A gene enrichment comparison analysis was performed for the canonical signaling pathways and molecular and cellular functions. The top 15 canonical signaling pathways (Fig. 3d) and molecular and cellular functions (Fig. 3e) are listed (comparison based on *p* value in Supplemental Figure 3). Strikingly, the activation status for each canonical signaling pathway varied greatly by chemical in HepG2 cells. Although DES and E_2_ shared the fewest genes in common, the heatmap of activation *z* scores for DES most closely resembled that of E_2_, demonstrating similar activation scores for approximately half of the canonical signaling pathways. The heatmaps for E_2_ and GEN were the most dissimilar, sharing similar activation scores for only 2 of the 15 top canonical signaling pathways. The top canonical pathways suggest a strong association with signaling related to cellular metabolism, regeneration, and repair [31, 32]. The gene enrichment data for the top molecular and cellular functions suggest that E_2_ and the xenoestrogens are potent regulators of cell death, survival, and movement in HepG2 cells. The molecular and cellular functions of TCDD and GEN most closely resembled those of E_2_, sharing similar predicted activation scores for cell viability, migration of tumor cell lines, and apoptosis.

To evaluate the predicted impact of these xenoestrogens on ER signaling, we utilized the IPA software to overlay the expression values of the differentially regulated genes on the “Estrogen Receptor Signaling” pathway, which highlights important components of ER signaling (Fig. 3f). Expression data of E_2_ treatment in HepG2 cells identified 14 significantly regulated molecules in the “Estrogen Receptor Signaling” pathway. EE2, TCDD, DES, BPA, and GEN also significantly regulated many molecules in the “Estrogen Receptor Signaling” pathway, supporting the potential of these chemicals to alter estrogen responsiveness. Certain molecules were commonly regulated by E_2_ and the xenoestrogens (*RNA Polymerase II*, *Taf*, *and TRAP/Media*), although the directionality of regulation was unique to the chemical and in some cases, the signaling molecule included both induced and repressed components (e.g., *TRAP/Media*). When controlling for cell-type, these results demonstrate that xenoestrogen exposure results in both overlapping and unique transcriptional responses, which likely support the divergent biological response to various xenoestrogens.

### The role of genetic sex in mediating the cellular response to EDCs

**Fig. 4:**
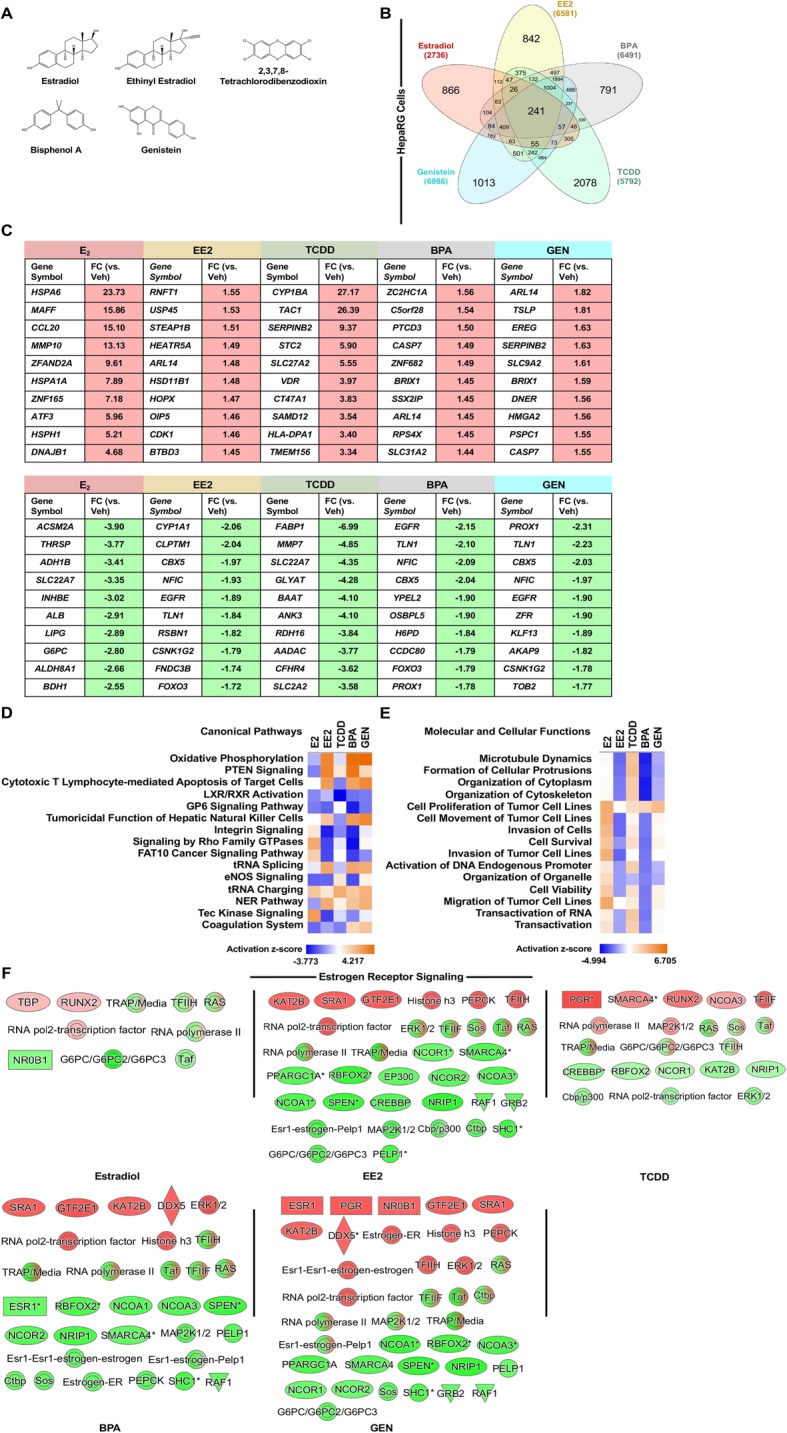
Comparison of transcriptional response to xenoestrogens in genetically female liver cells. Differentially expressed genes were determined for HepaRG cells treated with 1 μM ethinyl estradiol (EE2), 1 μM bisphenol A (BPA), 1 μM genistein (GEN), 10 nM 2,3,7,8-tetrachlorodibenzodioxin (TCDD) for 6 h or 30 μM estradiol (E_2_) for 12 h. **a** The chemical structures of E_2_, EE2, TCDD, BPA, and GEN were created using Chemdraw 18.0. **b** The xenoestrogen-regulated genes in HepaRG cells were compared by a Venn diagram to identify unique and commonly regulated genes. **c** The top 10 induced and repressed genes are listed by chemical. **d** A comparison analysis of the most significantly associated canonical pathways visualized by activation *z* score. **e** A comparison analysis of the top molecular and cellular functions utilizing the predicted activation *z* score. **f** Induced (red) and repressed (green) molecules associated with the “Estrogen Receptor Signaling” pathway visualized for each chemical

The sexual dimorphic response specific to ER action has been associated with the development of or protection from certain diseases (e.g., metabolic syndrome and autoimmune diseases) [33–36]. As such, animal studies have demonstrated that exogenous exposures that inappropriately alter ER signaling can increase the relative risk for these diseases [37–39]. To determine whether the genetic sex of a cell could influence the transcriptional response to the xenoestrogens, we repeated our analysis of differentially expressed genes in HepaRG cells, which were derived from the liver of a female patient [40]. Due to fewer available datasets, the analysis in HepaRG cells did not include DES treatment. Like HepG2 cells, the analysis in HepaRG cells included 6 h (1 μM BPA, 1 μM EE2, 1 μM GEN, and 10 nM TCDD), which was compared to a dataset from HepaRG cells treated for 12 h with E_2_ (Fig. 4a).

The Venn diagram of significantly regulated genes identified 241 genes that were common between E_2_ and the xenoestrogens (Fig. 4b). Compared to HepG2 cells, the xenoestrogens and E_2_ shared a greater number of commonly regulated genes in HepaRG cells. Commonly regulated genes were also apparent in the lists of top induced and repressed genes (Fig. 4c). For example, ADP ribosylation factor like GTPase 14 (*ARL14*), chromobox 5 (*CBX5*), nuclear factor I C (*NFIC*), epidermal growth factor receptor (*EGFR*), and talin 1 (*TLN1*) were included in the top regulated genes by EE2, BPA, and GEN. To determine how the activation status of predicted canonical signaling pathways and molecular and cellular functions would be altered by genetic sex, we evaluated gene annotations of the significantly regulated genes using the IPA software (comparison based on *p* value in Supplemental Figure 4). Interestingly, the top canonical signaling pathways regulated by E_2_ and the xenoestrogens were remarkably different in HepaRG cells compared to HepG2 cells, with only “Oxidative Phosphorylation” common to both cell lines (Fig. 4d). Although this pathway was highly regulated in HepaRG and HepG2 cells, the predicted activation status of “Oxidative Phosphorylation” for E_2_, EE2, BPA, and Gen was reversed in HepaRG cells compared to HepG2 cells. This suggests that the genetic sex of a cell has a strong influence on whether to activate or repress the transcription of target genes. The genetic sex of the cell also altered the type of canonical signaling pathways regulated, with a greater representation of immune-related pathways in the female HepaRG cells and more metabolism-related pathways in the male HepG2 cells. Compared to the canonical signaling pathways, the top molecular and cellular functions demonstrated slightly greater overlap between HepaRG and HepG2 cells, where 5 of the top 15 shared between the two cell lines (Fig. 4e). In HepaRG cells, there was a high degree of similarity between the activation status of functions regulated by E_2_ and TCDD and those regulated by EE2, BPA, and GEN when compared to the variability in activation status across chemicals in HepG2 cells.

The expression values of genes differentially regulated in HepaRG cells were overlaid on the “Estrogen Receptor Signaling” pathway (Fig. 4f). In HepaRG cells, E_2_ regulated fewer and different molecules compared to HepG2 cells. EE2, BPA, and GEN regulated many more molecules in the “Estrogen Receptor Signaling” pathway in HepaRG cells compared to HepG2 cells. These comparisons further illustrate how the genetic sex of a cell can result in regulatory specificity of a signaling pathway.

### Genetic sex influences the transcriptional response to genistein in hepatocytes

**Fig. 5:**
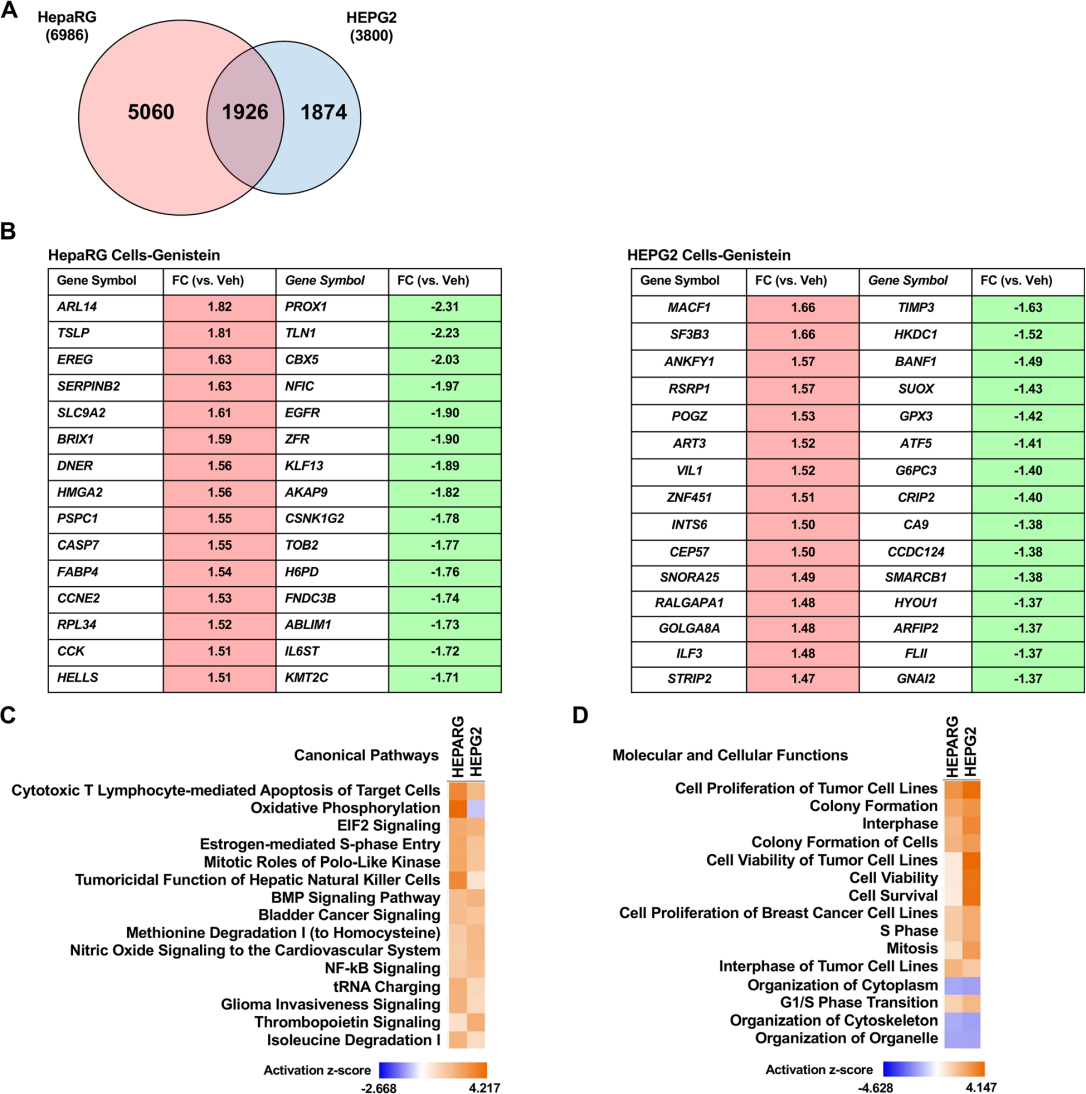
The transcriptional response of genetically female (HepaRG) and male (HepG2) hepatocytes to 1 μM GEN for 6 h. **a** A Venn diagram of similar and unique significantly altered genes (*p* < 0.05) in HepaRG and HepG2 cells. Top 10 induced or repressed genes in HepaRG (**b**) and HepG2 (**c**) cells. Top 10 predicted canonical signaling pathways and molecular and cellular functions by GEN in HepaRG (**d**) and HepG2 (**e**) cells. Induced or repressed molecules of the “Estrogen Receptor Signaling” pathway by GEN in HepaRG (**f**) and HepG2 (**g**) cells

As there was a large difference in the number of genes regulated by GEN in HepaRG and HepG2 cells, a direct comparison was conducted to evaluate the effect of genetic sex on the response to the phytoestrogen genistein. In both datasets, cells were treated for 6 h with 1 μM GEN. GEN treatment regulated almost twice as many genes in HepaRG cells compared to HepG2 cells (Fig. 5a). Moreover, less than 30% of the genes regulated by GEN in HepaRG cells were also regulated in HepG2 cells. Instead, 5060 genes were uniquely regulated by GEN in HepaRG cells. Not surprisingly, none of the top 15 induced or repressed genes were common between the two cell lines (Fig. 5b). Despite the stark differences in the number of genes regulated in hepatocyte cell lines of different sex, the activation status of the top predicted canonical signaling pathways (Fig. 5c) and molecular and cellular functions were very similar in the direct comparison (Fig. 5d) (comparison based on *p* value in Supplemental Figure 5). Notable differences between the two cell lines included the *p* value of the “Estrogen Receptor Signaling” pathway in HepaRG cells (2.08 × 10^−11^) and HepG2 cells (1.89 × 10^−1^), and the “Oxidative Phosphorylation” pathway was predicted to be activated in HepaRG cells and repressed in HepG2 cells. These analyses suggest that genes uniquely regulated by GEN in response to genetic sex may converge on similar cellular signaling pathways and functions.

## Discussion

The potential to interfere with endogenous cell signaling and contribute to pathophysiology has led to a myriad of studies that explored the mechanistic actions of xenoestrogens via gene expression studies. Many of these studies were limited to targeted cell-types, and the limitation of this approach is that conclusions deduced from one cell-type may not reflect the response in other cell-types, which express differing levels of the affected receptor, a cell-specific repertoire of transcriptional co-factors, and a unique chromatin environment. Our results suggest that in order to understand how xenoestrogens alter overall physiology, human cell lines originating from multiple organs and of different genetic sex must be utilized in gene expression studies to identify the cell-gene-environment interaction.

A total of 426 gene expression datasets were identified, although 88% of these datasets did not meet our inclusion criteria. The major constraint of comparing effects across a range of chemicals is the substantial differences in study design. For example, the identified gene expression studies included exposure lengths from 30 min to a 1-year chronic exposure. Moreover, the chemical concentration used for exposure ranged from 3 fm to 200 mM, which encompasses an order of magnitude for molarity from 10^−15^ to 10^−1^, respectively. Such a range provides the advantage of a broad analysis but also likely includes concentrations outside the range of expected exposure and into the spectrum of toxicity. Including RNA from cells that have entered the cell death pathway can confound results when evaluating intracellular signaling. For example, human serum BPA levels are estimated at 0.002–0.004 μM, but in some reported datasets, BPA was tested in HepG2 cells up to 200 mM [41, 42]. The half maximal concentration of BPA when evaluating 24 h cytotoxicity in HepG2 cells was demonstrated to be 261 μM, suggesting that gene expression studies performed with HepG2 cells treated for 48 h with millimolar concentrations may reflect the induction of cell death rather than endocrine disrupting activities [42]. Another important consideration when determining the physiological effect of EDCs is the in vivo metabolism of environmental chemicals into conjugated forms, which can alter their bioactivity [43]. In humans, BPA measured in urine and serum exists as an unconjugated form (20–32%), a glucuronidated form (46–57%), and as a sulfated form (7–34%) [44]. The glucuronidated form of BPA is biologically active, although its activity on the estrogen receptor differs from that of unconjugated BPA [45, 46]. Thus, in vitro studies replicating human exposure should consider how chemical metabolism impacts the relative levels of conjugated and unconjugated forms found in serum and adjust their treatment concentrations to reflect the bioavailable fraction of the studied chemical.

In this study, we found that the origin of the cell-type dictated the transcriptional response to the xenoestrogen genistein when controlling for concentration and exposure length. Context-driven plasticity is an important consideration for transcriptional regulatory factors and is based on the premise that the intracellular milieu of cofactors, epigenetically regulated chromatin environment, and differential usage of enhancer regions dictate the transcriptional response to a given stimulus [47, 48]. Thus, one potential source of transcriptional variation across cell-types is the relative expression of the estrogen receptors, which can mediate the effects of genistein [49]. Comparing the expression of ER in the three cell-types evaluated (liver, breast, and endometrium) using Human Protein Atlas demonstrated that *ERα* mRNA transcripts are highest in the endometrium, moderate in the breast, and relatively lower in the liver [50]. Protein levels of ERα followed a similar pattern, although ERα protein was not detected in the liver. The tissue expression of the estrogen receptor isoform *ERβ* is unique from *ERα* and reported as greater in the breast than the endometrium and relatively low in the liver. Differences in the abundance of ER isoforms provide one mechanism contributing to the unique transcriptional responses when cells originating from different organs are exposed to the same concentration of genistein. In addition to its estrogenic effects, genistein can inhibit the activity of receptor tyrosine kinases in a dose-dependent manner [51]. Thus, another source of transcriptional variation from genistein exposures could originate from the relative activity of receptor tyrosine kinase signaling in the studied cell-types. A third potential source of transcriptional variation between cell-types is the cell-specific chromatin environment. The accessibility of genomic regions, termed open chromatin, varies between cells, and subsequent activation of ER results in unique, cell-specific genomic interactions [52, 53]. When comparing the T47D breast cancer cell line and Ishikawa cells, only 19% of the ERα-binding sites are common between these two cell-types [54]. Finally enhancer regions, distal regulatory regions offering spatiotemporal control over transcriptional activity, differ between cell-types [48]. For example, a query of tumor-specific enhancer regions that regulate the expression of the MYC proto-oncogene identified seven up-stream regions in the colon cancer HCT-116 cell line and two enhancer regions down-stream of MYC in the leukemia K562 cell line [48]. Ultimately, transcriptional specificity across cell-types is a highly variable combination of transcription factor interactions with ligands and co-factors and the availability of DNA binding sites [55].

This study also found significant differences when comparing the transcriptional response to xenoestrogens within the same cell-type, which may reflect properties specific to the xenoestrogen. The xenoestrogens investigated are largely capable of binding ER, although these chemicals demonstrate unique binding affinities for the two ER isoforms [56–59]. For example, genistein has a stronger affinity for ERβ than ERα, while BPA has a relatively weak affinity for both ER isoforms. Moreover, the xenoestrogens exert differential effects on ER activity. For example, BPA is a complete ERβ antagonist, while diethylstilbestrol has agonistic effects, and genistein is partially agonistic [56–60]. Some of the xenoestrogens evaluated also have documented effects in the absence of ER through binding other receptors. For example, TCDD elicits its xenoestrogenic effects through binding AhR, rather than either ER isoform [61, 62]. Furthermore, genistein, BPA, and diethylstilbestrol have known affinities for the estrogen-related receptors (ERRs), orphan nuclear receptors that do not appear to bind any estrogen [63–65]. The unique binding affinities and agonistic/antagonistic relationships of these xenoestrogens with estrogen and non-estrogen receptors likely contribute to variability in the differentially regulated genes when comparing xenoestrogens within one cell type.

Genetic sex is also increasingly being recognized as an important mediating factor in the cellular response, leading to a recent overhaul in the experimental design of all NIH-sponsored studies (reviewed in [66–68]). Although the HepG2 and HepaRG cell lines both represent human hepatoma cells, the same environmental exposure resulted in distinct differentially expressed genes in the male HepG2 compared to the female HepaRG cells. We found that only 28% of the genes differentially regulated in HepaRG cells was also significantly regulated in the HepG2 cells. These findings are similar to those reported in a study by Jennen et al., where chemical exposure largely produced HepG2- and HepaRG-unique genes [25]. Sexual dimorphic responses have also been described in primary hepatocytes [69]. Both our current findings and previous studies highlight a need for addressing sex as a source of a variation beyond whole-organismal findings and extending the scope of sex-based differences to the cellular level.

Due to our strict criteria for inclusion in analysis, potential limitations exist in our study. For example, the comparison of effects across cell-types was limited to three cell lines based on available datasets. Moreover, the restriction on concentration used eliminated all datasets from analysis for certain chemicals. Additionally, reposited data represent gene expression studies that employed various array platforms, which may introduce some variation in represented genes. Nonetheless, the identified datasets largely originated from the same gene expression super series (GSE69851), which exclusively utilized the Affymetrix Human Genome U219 Array. This array platform was used in 10 out of 13 datasets used for analysis. The remaining three datasets utilized the Affymetrix Human Genome U133 Plus 2.0 Array. The primary difference between these two Affymetrix arrays is an increase in the total probe sets included in the U219 array. However, greater than 90% of the probe sets on the U219 Array are shared by the U133 Plus 2.0 Array.

## Conclusions

The findings presented here suggest that while xenoestrogens belong to the same EDC subgroup, they exert diverse effects on transcription. Moreover, the effect on transcription is highly regulated by the type of cell exposed. We also provide evidence that genetic sex can mediate how an EDC alters gene expression. This study highlights the importance of evaluating multiple cell-types when exploring the transcriptional response to EDCs. Conclusions based on a single cell-type or representing only one genetic sex may not accurately reflect the ability of a chemical to interfere with the normal physiology of endocrine tissues throughout the body. Thus, future studies can broaden the translational relevance of gene-environment interaction studies by considering the effect of EDCs across a range of cell-types.

## Supplementary information

**Additional file 1:** Supplemental Data

**Additional file 2:** Supplemental Figures

## Data Availability

The datasets analyzed as part of this study are publicly available via https://www.ncbi.nlm.nih.gov/geo/ and https://www.ebi.ac.uk/arrayexpress/.
