# Supplementary material for: Gene X environment: the cellular environment governs the transcriptional response to environmental chemicals
Source: Hum Genomics. 2020 May 24;14:19. doi: 10.1186/s40246-020-00269-1 (PMC7247264; doi:10.1186/s40246-020-00269-1)
Supplement: Supplementary file 1 — Additional file 1: Supplemental Data [file 40246_2020_269_MOESM1_ESM.docx]

SUPPLEMENTAL DATA

Supplemental Figure 1. Principle Component Analysis (PCA) of gene expression data by xenoestrogen exposure and cell type. Each analysis included the appropriate vehicle as determined by experimental accession information.

Supplemental Figure 2. Transcriptional response to genistein across cell-types. Comparison analysis of the most significantly regulated Canonical Pathways and Molecular and Cellular Functions ranked by p-value.

Supplemental Figure 3. Comparison of the transcriptional response to xenoestrogens in HepG2 cells. Comparison analysis of the most significantly regulated Canonical Pathways and Molecular and Cellular Functions ranked by p-value.

Supplemental Figure 4. Comparison of the transcriptional response to xenoestrogens in HepaRG cells. Comparison analysis of the most significantly regulated Canonical Pathways and Molecular and Cellular Functions ranked by p-value.

Supplemental Figure 5. Comparing the transcriptional response of genetically female (HepaRG) and male (HepG2) hepatocytes to genistein. Comparison analysis of the most significantly regulated Canonical Pathways and Molecular and Cellular Functions ranked by p-value.

Supplemental Table 1. Identified Gene Expression Arrays by Xenoestrogen Classified by Cell Type, Chemical Treatment, Duration of Exposure, Chemical Concentration, Number of Experimental Replicates, and Genetic Sex of Cell Line.

| **Chemical** | **Cell Line Type** | **Tissue of origin** | **Time Course (h)** | **Dose** | **Replicate #** | **Sex** | **Accession # (*used in analysis)** |
| --- | --- | --- | --- | --- | --- | --- | --- |
| Atrazine | HepG2 | Liver | 24 | 700uM | 3 | M | E-MTAB-3509 |
| Atrazine | MCF-7 | Breast | 1 | 10uM | 2 | F | GSE16881 |
| Atrazine | MCF-7 | Breast | 4 | 10uM | 2 | F | GSE16881 |
| Atrazine | MCF-7 | Breast | 48 | 10uM | 2 | F | GSE16881 |
| Atrazine | MCF-7 | Breast | 1 | 40uM | 2 | F | GSE16881 |
| Atrazine | MCF-7 | Breast | 4 | 40uM | 2 | F | GSE16881 |
| Atrazine | MCF-7 | Breast | 48 | 40uM | 2 | F | GSE16881 |
| Atrazine | SH-SY5Y | Bone marrow | 24 | 463.5uM | 3 | F | GSE24499 |
| BPA | Adipocytes of lean children | Inguinal fat | 24 | 10nM | 5 | M | GSE58516 |
| BPA | ER:PRL-HeLa cells | Cervix | 4 | 10mM | 1 | F | GSE22941 |
| BPA | Fibroblast | Skin | 24 | 10nM | 3 | M | GSE35034 |
| BPA | HEEC | Endometrial | 24 | 50uM | 5 | F | GSE10802 |
| BPA | HEK293 | Kidney | 48 | 1uM | 1 | F | E-MTAB-1959 |
| BPA | HepaRG | Liver | 6 | 100uM | 3 | F | GSE69844 |
| BPA | HepaRG | Liver | 6 | 10uM | 3 | F | GSE69844 |
| BPA | HepaRG | Liver | 6 | 1uM | 3 | F | GSE69844* |
| BPA | HepG2 | Liver | 6 | 100uM | 3 | M | GSE69850 |
| BPA | HepG2 | Liver | 6 | 10uM | 3 | M | GSE69850 |
| BPA | HepG2 | Liver | 6 | 1uM | 3 | M | GSE69850* |
| BPA | HepG2 | Liver | 48 | 200mM | 4 | M | E-TOXM-31 |
| BPA | HepG2 | Liver | 48 | 200mM | 4 | M | E-TOXM-31 |
| BPA | HepG2 | Liver | 48 | 20mM | 3 | M | E-TOXM-31 |
| BPA | HepG2 | Liver | 48 | 20mM | 4 | M | E-TOXM-31 |
| BPA | HOS | Osteosarcoma | 8 | 10nM | 3 | F | GSE50527 |
| BPA | HOS | Osteosarcoma | 2160 | 10nM | 3 | F | GSE50527 |
| BPA | Ishikawa | Endometrial | 8 | 100nM | 2 | F | GSE38234 |
| BPA | Ishikawa | Endometrium | 8 | 100nM | 2 | F | GSE93437 |
| BPA | Ishikawa | Endometrium | 8 | 100pM | 4 | F | GSE17624 |
| BPA | Ishikawa | Endometrium | 24 | 100pM | 4 | F | GSE17624 |
| BPA | Ishikawa | Endometrium | 48 | 100pM | 4 | F | GSE17624 |
| BPA | Ishikawa | Endometrium | 6 | 100uM | 3 | F | GSE69849 |
| BPA | Ishikawa | Endometrium | 8 | 10nM | 4 | F | GSE17624 |
| BPA | Ishikawa | Endometrium | 24 | 10nM | 4 | F | GSE17624 |
| BPA | Ishikawa | Endometrium | 48 | 10nM | 4 | F | GSE17624 |
| BPA | Ishikawa | Endometrium | 6 | 10uM | 3 | F | GSE69849 |
| BPA | Ishikawa | Endometrium | 8 | 1pM | 4 | F | GSE17624 |
| BPA | Ishikawa | Endometrium | 24 | 1pM | 4 | F | GSE17624 |
| BPA | Ishikawa | Endometrium | 48 | 1pM | 4 | F | GSE17624 |
| BPA | Ishikawa | Endometrium | 6 | 1uM | 3 | F | GSE69849 |
| BPA | Ishikawa | Endometrium | 8 | 1uM | 4 | F | GSE17624 |
| BPA | Ishikawa | Endometrium | 24 | 1uM | 4 | F | GSE17624 |
| BPA | Ishikawa | Endometrium | 48 | 1uM | 4 | F | GSE17624 |
| BPA | MCF-10F | Breast | 336 | 10uM | 2 | F | GSE26884 |
| BPA | MCF-10F | Breast | 336 | 10uM | 4 | F | GSE32158 |
| BPA | MCF-10F | Breast | 336 | 1uM | 2 | F | GSE26884 |
| BPA | MCF-10F | Breast | 336 | 1uM | 2 | F | GSE32158 |
| BPA | MCF-7 | Breast | 48 | 1.56uM | 2 | F | GSE50705 |
| BPA | MCF-7 | Breast | 48 | 1.95nM | 3 | F | GSE50705 |
| BPA | MCF-7 | Breast | 48 | 1.95pM | 2 | F | GSE50705 |
| BPA | MCF-7 | Breast | 6 | 100uM | 3 | F | GSE69845 |
| BPA | MCF-7 | Breast | 48 | 100uM | 1 | F | GSE50705 |
| BPA | MCF-7 | Breast | 1 | 10uM | 2 | F | GSE16881 |
| BPA | MCF-7 | Breast | 4 | 10uM | 2 | F | GSE16881 |
| BPA | MCF-7 | Breast | 6 | 10uM | 3 | F | GSE69845 |
| BPA | MCF-7 | Breast | 18 | 10uM | 1 | F | GSE33481 |
| BPA | MCF-7 | Breast | 48 | 10uM | 2 | F | GSE16881 |
| BPA | MCF-7 | Breast | 48 | 12.5uM | 4 | F | GSE50705 |
| BPA | MCF-7 | Breast | 48 | 125nM | 4 | F | GSE50705 |
| BPA | MCF-7 | Breast | 48 | 125pM | 1 | F | GSE50705 |
| BPA | MCF-7 | Breast | 48 | 15.6nM | 6 | F | GSE50705 |
| BPA | MCF-7 | Breast | 1 | 1uM | 2 | F | GSE16881 |
| BPA | MCF-7 | Breast | 4 | 1uM | 2 | F | GSE16881 |
| BPA | MCF-7 | Breast | 6 | 1uM | 3 | F | GSE69845 |
| BPA | MCF-7 | Breast | 24 | 1uM | 2 | F | GSE45721 |
| BPA | MCF-7 | Breast | 48 | 1uM | 2 | F | GSE16881 |
| BPA | MCF-7 | Breast | 48 | 1uM | 6 | F | GSE50705 |
| BPA | MCF-7 | Breast | 48 | 250nM | 6 | F | GSE50705 |
| BPA | MCF-7 | Breast | 48 | 250pM | 3 | F | GSE50705 |
| BPA | MCF-7 | Breast | 48 | 25uM | 3 | F | GSE50705 |
| BPA | MCF-7 | Breast | 48 | 2uM | 2 | F | GSE50705 |
| BPA | MCF-7 | Breast | 48 | 3.125uM | 3 | F | GSE50705 |
| BPA | MCF-7 | Breast | 48 | 3.12uM | 1 | F | GSE50705 |
| BPA | MCF-7 | Breast | 48 | 3.91nM | 1 | F | GSE50705 |
| BPA | MCF-7 | Breast | 48 | 3.91pM | 1 | F | GSE50705 |
| BPA | MCF-7 | Breast | 48 | 31.25nM | 2 | F | GSE50705 |
| BPA | MCF-7 | Breast | 48 | 31.25pM | 2 | F | GSE50705 |
| BPA | MCF-7 | Breast | 48 | 398.6nM | 3 | F | GSE86472 |
| BPA | MCF-7 | Breast | 48 | 400nM | 3 | F | GSE85350 |
| BPA | MCF-7 | Breast | 48 | 4uM | 2 | F | GSE50705 |
| BPA | MCF-7 | Breast | 48 | 500nM | 4 | F | GSE50705 |
| BPA | MCF-7 | Breast | 48 | 500pM | 1 | F | GSE50705 |
| BPA | MCF-7 | Breast | 8760 | 50nM | 2 | F | GSE59345 |
| BPA | MCF-7 | Breast | 48 | 50uM | 4 | F | GSE50705 |
| BPA | MCF-7 | Breast | 48 | 6.25uM | 2 | F | GSE50705 |
| BPA | MCF-7 | Breast | 48 | 62.5nM | 6 | F | GSE50705 |
| BPA | MCF-7 | Breast | 48 | 62.5pM | 1 | F | GSE50705 |
| BPA | MCF-7 | Breast | 48 | 7.81nM | 2 | F | GSE50705 |
| BPA | MCF-7 | Breast | 48 | 7.81pM | 1 | F | GSE50705 |
| BPA | MCF-7 | Breast | 48 | 8uM | 2 | F | GSE50705 |
| BPA | MCF-7 | Breast | 48 | N/A | 3 | F | GSE87701 |
| BPA | MCF-7/BUS | Breast | 48 | 10nM | 3 | F | GSE5200 |
| BPA | Preadipocytes | Inguinal fat | 48 | 25uM | 5 | F | GSE56085 |
| BPA | Preadipocytes | Inguinal fat | 48 | 50uM | 5 | F | GSE56085 |
| BPA | Prostate epithelial cells | Prostate | 168 | 1uM | 3 | M | GSE62953 |
| BPA | Prostate epithelial cells | Prostate | 168 | 200nM | 3 | M | GSE62953 |
| BPA | Prostate epithelial cells | Prostate | 168 | 50uM | 5 | M | GSE62953 |
| BPA | SKOV3 | Ovarian | 24 | 100nM | 3 | F | E-MTAB-6295 |
| BPA | SKOV3 | Ovarian | 24 | 10nM | 3 | F | E-MTAB-6295 |
| BPA | T-47D | Breast | 4 | 100nM | 2 | F | GSE93441 |
| BPA | T-47D | Breast | 8 | 100nM | 2 | F | GSE38234 |
| BPA | White subcuteaneous adipocyte | Subcuteanous fat | 240 | 10nM | 3 | F | GSE98682 |
| BPA | White subcuteaneous adipocyte | Subcuteanous fat | 240 | 10uM | 3 | F | GSE98682 |
| BPAF | HOS | Osteosarcoma | 8 | 10nM | 3 | F | GSE50527 |
| BPAF | HOS | Osteosarcoma | 2160 | 10nM | 3 | F | GSE50527 |
| BPAF | MCF-7 | Breast | 48 | 80nM | 3 | F | GSE85350 |
| BPAF | MCF-7 | Breast | 48 | N/A | 3 | F | GSE87701 |
| BPS | HOS | Osteosarcoma | 8 | 10nM | 3 | F | GSE50527 |
| BPS | HOS | Osteosarcoma | 2160 | 10nM | 3 | F | GSE50527 |
| BPS | MCF-7 | Breast | 48 | 1.5uM | 3 | F | GSE85350 |
| BPS | White subcuteaneous adipocyte | Subcuteanous fat | 240 | 10nM | 3 | F | GSE98680 |
| BPS | White subcuteaneous adipocyte | Subcuteanous fat | 240 | 10uM | 3 | F | GSE98680 |
| Daidzein | HDFa | Skin | 24 | 100uM | 3 | N/A | GSE43692 |
| Daidzein | HDFa | Skin | 48 | 100uM | 3 | N/A | GSE43692 |
| Daidzein | HDFa | Skin | 24 | 60uM | 3 | N/A | GSE43692 |
| Daidzein | HDFa | Skin | 48 | 60uM | 3 | N/A | GSE43692 |
| Daidzein | MCF-7 | Breast | 48 | 1uM | 4 | F | GSE50705 |
| Daidzein | MCF-7 | Breast | 48 | 2uM | 4 | F | GSE50705 |
| Daidzein | MCF-7 | Breast | 48 | 3uM | 4 | F | GSE50705 |
| Daidzein | MCF-7 | Breast | 48 | 4uM | 4 | F | GSE50705 |
| Daidzein | MCF-7 | Breast | 48 | 500nM | 4 | F | GSE50705 |
| Daidzein | MCF-7/BUS | Breast | 48 | 1uM | 3 | F | GSE5200 |
| DDT | HEEC | Uterus | 24 | 50uM | 4 | F | GSE9252 |
| DDT | HepG2 | Liver | 24 | 80uM | 3 | M | GSE28878 |
| DDT | HepG2 | Liver | 48 | 80uM | 3 | M | GSE28878 |
| DDT | MCF-7 | Breast | 18 | 10uM | 1 | F | GSE33481 |
| DDT | MCF-7 | Breast | 1 | 1uM | 2 | F | GSE16881 |
| DDT | MCF-7 | Breast | 4 | 1uM | 2 | F | GSE16881 |
| DDT | MCF-7 | Breast | 48 | 1uM | 2 | F | GSE16881 |
| DDT | MCF-7 | Breast | 1 | 5uM | 2 | F | GSE16881 |
| DDT | MCF-7 | Breast | 4 | 5uM | 2 | F | GSE16881 |
| DDT | MCF-7 | Breast | 48 | 5uM | 2 | F | GSE16881 |
| DEHP | HepaRG | Liver | 24 | 10mM | 3 | F | GSE48990 |
| DEHP | HepaRG | Liver | 72 | 10mM | 3 | F | GSE48990 |
| DEHP | HepG2 | Liver | 12 | 10mM | 3 | M | GSE28878 |
| DEHP | HepG2 | Liver | 24 | 10mM | 3 | M | GSE28878 |
| DEHP | HepG2 | Liver | 48 | 10mM | 3 | M | GSE28878 |
| DEHP | HepG2 | Liver | 2160 | 250pM | 1 | M | GSE83321 |
| DEHP | Ishikawa | uterus | 48 | 50uM | 2 | F | GSE43653 |
| DEHP | Jurkat | T cell | 6 | 300uM | 4 | M | GSE46909 |
| DEHP | MCF-7 | Breast | 1 | 10uM | 2 | F | GSE16881 |
| DEHP | MCF-7 | Breast | 4 | 10uM | 2 | F | GSE16881 |
| DEHP | MCF-7 | Breast | 48 | 10uM | 2 | F | GSE16881 |
| DEHP | MCF-7 | Breast | 1 | 5uM | 2 | F | GSE16881 |
| DEHP | MCF-7 | Breast | 4 | 5uM | 2 | F | GSE16881 |
| DEHP | MCF-7 | Breast | 48 | 5uM | 2 | F | GSE16881 |
| DES | HepG2 | Liver | 12 | 5uM | 3 | M | GSE28878* |
| DES | HepG2 | Liver | 24 | 5uM | 3 | M | GSE28878 |
| DES | HepG2 | Liver | 48 | 5uM | 3 | M | GSE28878 |
| DES | KhES-3 | Embryonic Stem Cell | 48 | N/A | 1 | M | GSE60154 |
| DES | MCF-7 | Breast | 48 | 100fM | 5 | F | GSE50705 |
| DES | MCF-7 | Breast | 48 | 10fM | 5 | F | GSE50705 |
| DES | MCF-7 | Breast | 48 | 300fM | 5 | F | GSE50705 |
| DES | MCF-7 | Breast | 48 | 30fM | 5 | F | GSE50705 |
| DES | MCF-7 | Breast | 48 | 3fM | 5 | F | GSE50705 |
| DES | TK6 | Spleen | 4 | 40uM | 1 | M | GSE107162 |
| EE2 | HepaRG | Liver | 6 | 100nM | 3 | F | GSE69844 |
| EE2 | HepaRG | Liver | 6 | 10nM | 3 | F | GSE69844 |
| EE2 | HepaRG | Liver | 6 | 1uM | 3 | F | GSE69844* |
| EE2 | HepG2 | Liver | 6 | 100nM | 3 | M | GSE69850 |
| EE2 | HepG2 | Liver | 6 | 10nM | 3 | M | GSE69850 |
| EE2 | HepG2 | Liver | 6 | 1uM | 3 | M | GSE69850* |
| EE2 | Ishikawa | Endometrium | 72 | 100fM | 6 | F | GSE84711 |
| EE2 | Ishikawa | Endometrium | 6 | 100nM | 3 | F | GSE69849 |
| EE2 | Ishikawa | Endometrium | 72 | 100pM | 6 | F | GSE84711 |
| EE2 | Ishikawa | Endometrium | 6 | 10nM | 3 | F | GSE69849 |
| EE2 | Ishikawa | Endometrium | 72 | 10pM | 6 | F | GSE84711 |
| EE2 | Ishikawa | Endometrium | 72 | 1nM | 6 | F | GSE84711 |
| EE2 | Ishikawa | Endometrium | 72 | 1pM | 6 | F | GSE84711 |
| EE2 | Ishikawa | Endometrium | 6 | 1uM | 3 | F | GSE69849 |
| EE2 | Ishikawa | Endometrium | 72 | 300fM | 6 | F | GSE84711 |
| EE2 | Ishikawa | Endometrium | 72 | 3pM | 6 | F | GSE84711 |
| EE2 | MCF-7 | Breast | 6 | 100nM | 3 | F | GSE69845 |
| EE2 | MCF-7 | Breast | 48 | 100pM | 5 | F | GSE50705 |
| EE2 | MCF-7 | Breast | 6 | 10nM | 3 | F | GSE69845 |
| EE2 | MCF-7 | Breast | 48 | 10pM | 5 | F | GSE50705 |
| EE2 | MCF-7 | Breast | 48 | 1pM | 5 | F | GSE50705 |
| EE2 | MCF-7 | Breast | 6 | 1uM | 3 | F | GSE69845 |
| EE2 | MCF-7 | Breast | 48 | 30pM | 5 | F | GSE50705 |
| EE2 | MCF-7 | Breast | 48 | 3pM | 5 | F | GSE50705 |
| EE2 | MCF-7 | Breast | 48 | 60pM | 5 | F | GSE50705 |
| EE2 | Primary hepatocyte | Liver | 8 | N/A | 2 | M | MTAB-798 |
| EE2 | Primary hepatocyte | Liver | 24 | N/A | 2 | M | MTAB-798 |
| Estrone | A673 | Muscle | 6 | 30nM | 1 | F | GSE119291 |
| Estrone | AGS | Stomach | 6 | 30nM | 1 | F | GSE119291 |
| Estrone | HepG2 | Liver | 6 | 30nM | 1 | M | GSE119291 |
| Estrone | hES | Embryonic Stem Cell | 72 | 100nM | 1 | F | GSE58591 |
| Estrone | hES | Embryonic Stem Cell | 72 | 100nM | 1 | M | GSE58591 |
| Estrone | hES | Embryonic Stem Cell | 264 | 100nM | 2 | F | GSE58591 |
| Estrone | hES | Embryonic Stem Cell | 264 | 100nM | 2 | M | GSE58591 |
| Estrone | HT29 | Colon | 6 | 30nM | 1 | F | GSE119291 |
| Estrone | MCF-7 | Breast | 6 | 30nM | 1 | F | GSE119291 |
| Estrone | SH-SY5Y | Bone marrow | 6 | 30nM | 1 | F | GSE119291 |
| Estrone | VCAP | Prostate | 6 | 30nM | 1 | M | GSE119291 |
| Genistein | ARCaP-E | Prostate | 212 | 20uM | 3 | M | GSE35242 |
| Genistein | ARCaP-M | Prostate | 212 | 20uM | 3 | M | GSE35242 |
| Genistein | CFBE41o-ΔF508 | Bronchus | 24 | 10uM | 2 | N/A | GSE67698 |
| Genistein | DU145 | Prostate | 96 | 25uM | 1 | M | GSE46757 |
| Genistein | FTC-238/hrTPO/RSK008 | Thyroid | 48 | 1uM | 2 | M | GSE30961 |
| Genistein | HaCaT | Skin | 24 | 100uM | 3 | M | GSE60971 |
| Genistein | HaCaT | Skin | 48 | 100uM | 3 | M | GSE60971 |
| Genistein | HaCaT | Skin | 24 | 30uM | 3 | M | GSE60971 |
| Genistein | HaCaT | Skin | 48 | 30uM | 3 | M | GSE60971 |
| Genistein | HaCaT | Skin | 24 | 60uM | 3 | M | GSE60971 |
| Genistein | HaCaT | Skin | 48 | 60uM | 3 | M | GSE60971 |
| Genistein | HDFa | Skin | 1 | 100uM | 5 | Lot Specific | GSE34074 |
| Genistein | HDFa | Skin | 24 | 100uM | 5 | Lot Specific | GSE34074 |
| Genistein | HDFa | Skin | 48 | 100uM | 5 | Lot Specific | GSE34074 |
| Genistein | HDFa | Skin | 1 | 30uM | 5 | Lot Specific | GSE34074 |
| Genistein | HDFa | Skin | 24 | 30uM | 5 | Lot Specific | GSE34074 |
| Genistein | HDFa | Skin | 48 | 30uM | 5 | Lot Specific | GSE34074 |
| Genistein | HDFa | Skin | 1 | 60uM | 3 | Lot Specific | GSE34074 |
| Genistein | HDFa | Skin | 24 | 60uM | 3 | Lot Specific | GSE34074 |
| Genistein | HDFa | Skin | 48 | 60uM | 3 | Lot Specific | GSE34074 |
| Genistein | HepaRG | Liver | 6 | 100uM | 3 | F | GSE69844 |
| Genistein | HepaRG | Liver | 6 | 10uM | 3 | F | GSE69844 |
| Genistein | HepaRG | Liver | 6 | 1uM | 3 | F | GSE69844* |
| Genistein | HepG2 | Liver | 6 | 100uM | 3 | M | GSE69850 |
| Genistein | HepG2 | Liver | 6 | 10uM | 3 | M | GSE69850 |
| Genistein | HepG2 | Liver | 18 | 10uM | 3 | M | GSE40069 |
| Genistein | HepG2 | Liver | 6 | 1uM | 3 | M | GSE69850* |
| Genistein | iPS-derived cadiomyocytes | Heart | 12 | 1uM | 1 | N/A | GSE89972 |
| Genistein | Ishikawa | Endometrium | 4 | 100nM | 2 | F | GSE93440 |
| Genistein | Ishikawa | Endometrium | 8 | 100nM | 2 | F | GSE38234 |
| Genistein | Ishikawa | Endometrium | 8 | 100nM | 4 | F | GSE71717 |
| Genistein | Ishikawa | Endometrium | 24 | 100nM | 4 | F | GSE71717 |
| Genistein | Ishikawa | Endometrium | 48 | 100nM | 4 | F | GSE71717 |
| Genistein | Ishikawa | Endometrium | 6 | 100uM | 3 | F | GSE69849 |
| Genistein | Ishikawa | Endometrium | 8 | 10pM | 4 | F | GSE71717 |
| Genistein | Ishikawa | Endometrium | 24 | 10pM | 4 | F | GSE71717 |
| Genistein | Ishikawa | Endometrium | 48 | 10pM | 4 | F | GSE71717 |
| Genistein | Ishikawa | Endometrium | 6 | 10uM | 3 | F | GSE69849 |
| Genistein | Ishikawa | Endometrium | 8 | 10uM | 4 | F | GSE71717 |
| Genistein | Ishikawa | Endometrium | 24 | 10uM | 4 | F | GSE71717 |
| Genistein | Ishikawa | Endometrium | 48 | 10uM | 4 | F | GSE71717 |
| Genistein | Ishikawa | Endometrium | 8 | 1nM | 4 | F | GSE71717 |
| Genistein | Ishikawa | Endometrium | 24 | 1nM | 4 | F | GSE71717 |
| Genistein | Ishikawa | Endometrium | 48 | 1nM | 4 | F | GSE71717 |
| Genistein | Ishikawa | Endometrium | 6 | 1uM | 3 | F | GSE69849* |
| Genistein | LNCaP | Prostrate | 4 | 15uM | 1 | M | GSE24796 |
| Genistein | LNCaP | Prostrate | 8 | 15uM | 1 | M | GSE24796 |
| Genistein | LNCaP | Prostrate | 12 | 15uM | 1 | M | GSE24796 |
| Genistein | LNCaP | Prostrate | 18 | 15uM | 1 | M | GSE24796 |
| Genistein | LNCaP | Prostrate | 24 | 15uM | 1 | M | GSE24796 |
| Genistein | LNCaP | Prostrate | 36 | 15uM | 1 | M | GSE24796 |
| Genistein | LNCaP | Prostrate | 48 | 15uM | 1 | M | GSE24796 |
| Genistein | LNCaP | Prostrate | 4 | 2uM | 1 | M | GSE24796 |
| Genistein | LNCaP | Prostrate | 8 | 2uM | 1 | M | GSE24796 |
| Genistein | LNCaP | Prostrate | 12 | 2uM | 1 | M | GSE24796 |
| Genistein | LNCaP | Prostrate | 18 | 2uM | 1 | M | GSE24796 |
| Genistein | LNCaP | Prostrate | 24 | 2uM | 1 | M | GSE24796 |
| Genistein | LNCaP | Prostrate | 36 | 2uM | 1 | M | GSE24796 |
| Genistein | LNCaP | Prostrate | 48 | 2uM | 1 | M | GSE24796 |
| Genistein | MCF-7 | Breast | 48 | 100nM | 1 | F | GSE50705 |
| Genistein | MCF-7 | Breast | 6 | 100uM | 3 | F | GSE69845 |
| Genistein | MCF-7 | Breast | 4 | 10uM | 2 | F | GSE16881 |
| Genistein | MCF-7 | Breast | 6 | 10uM | 4 | F | GSE5258 |
| Genistein | MCF-7 | Breast | 6 | 10uM | 3 | F | GSE69845 |
| Genistein | MCF-7 | Breast | 48 | 10uM | 2 | F | GSE16881 |
| Genistein | MCF-7 | Breast | 48 | 10uM | 3 | F | GSE50705 |
| Genistein | MCF-7 | Breast | 48 | 12uM | 3 | F | GSE50705 |
| Genistein | MCF-7 | Breast | 48 | 16uM | 4 | F | GSE50705 |
| Genistein | MCF-7 | Breast | 1 | 1uM | 2 | F | GSE16881 |
| Genistein | MCF-7 | Breast | 4 | 1uM | 2 | F | GSE16881 |
| Genistein | MCF-7 | Breast | 6 | 1uM | 2 | F | GSE5258 |
| Genistein | MCF-7 | Breast | 6 | 1uM | 3 | F | GSE69845* |
| Genistein | MCF-7 | Breast | 24 | 1uM | 2 | F | GSE45721 |
| Genistein | MCF-7 | Breast | 48 | 1uM | 2 | F | GSE16881 |
| Genistein | MCF-7 | Breast | 48 | 1uM | 4 | F | GSE50705 |
| Genistein | MCF-7 | Breast | 48 | 250nM | 3 | F | GSE50705 |
| Genistein | MCF-7 | Breast | 48 | 2uM | 7 | F | GSE50705 |
| Genistein | MCF-7 | Breast | 48 | 3uM | 7 | F | GSE50705 |
| Genistein | MCF-7 | Breast | 48 | 4uM | 7 | F | GSE50705 |
| Genistein | MCF-7 | Breast | 48 | 500nM | 7 | F | GSE50705 |
| Genistein | MCF-7 | Breast | 48 | 50nM | 3 | F | GSE50705 |
| Genistein | MCF-7 | Breast | 8760 | 50nM | 2 | F | GSE59345 |
| Genistein | MCF-7 | Breast | 48 | 6uM | 3 | F | GSE50705 |
| Genistein | MCF-7 | Breast | 48 | 750nM | 3 | F | GSE50705 |
| Genistein | MCF-7 | Breast | 48 | 8uM | 7 | F | GSE50705 |
| Genistein | MCF-7 (control adenovirus) | Breast | 24 | 1uM | 2 | F | GSE56066 |
| Genistein | MCF-7 (Erb-expressing adenovirus) | Breast | 24 | 1uM | 2 | F | GSE56066 |
| Genistein | MCF-7/BUS | Breast | 48 | 10uM | 3 | F | GSE5200 |
| Genistein | MCF-7/BUS | Breast | 48 | 3uM | 3 | F | GSE5200 |
| Genistein | PC3 | Prostate | 6 | 10uM | 1 | M | GSE5258 |
| Genistein | PC3 | prostate | 144 | 20uM | 1 | M | E-MEXP-2034 |
| Genistein | PC3 | Prostate | 96 | 25uM | 1 | M | GSE47657 |
| Genistein | PCa | Prostate | 672 | N/A | 10 | M | GSE128339 |
| Genistein | T-47D | Breast | 4 | 100nM | 2 | F | GSE93444 |
| Genistein | T-47D | Breast | 8 | 100nM | 2 | F | GSE38234 |
| Genistein | TK6 | Spleen | 4 | 20uM | 2 | M | GSE107162 |
| Genistein | UtLM | Uterus | 24 | 185uM | 6 | F | GSE19477 |
| MOC | HepG2 | Liver | 24 | 20uM | 4 | M | E-TOXM-31 |
| MOC | HepG2 | Liver | 48 | 20uM | 4 | M | E-TOXM-31 |
| MOC | HepG2 | Liver | 24 | 2uM | 2 | M | E-TOXM-31 |
| MOC | HepG2 | Liver | 48 | 2uM | 4 | M | E-TOXM-31 |
| MOC | MCF-7 | Breast | 1 | 15uM | 2 | F | GSE16881 |
| MOC | MCF-7 | Breast | 4 | 15uM | 2 | F | GSE16881 |
| MOC | MCF-7 | Breast | 48 | 15uM | 2 | F | GSE16881 |
| MOC | MCF-7 | Breast | 1 | 5uM | 2 | F | GSE16881 |
| MOC | MCF-7 | Breast | 4 | 5uM | 2 | F | GSE16881 |
| MOC | MCF-7 | Breast | 48 | 5uM | 2 | F | GSE16881 |
| NP | MCF-7 | Breast | 48 | 1.95nM | 1 | F | GSE50705 |
| NP | MCF-7 | Breast | 1 | 10uM | 2 | F | GSE16881 |
| NP | MCF-7 | Breast | 48 | 10uM | 2 | F | GSE16881 |
| NP | MCF-7 | Breast | 48 | 12.5uM | 3 | F | GSE50705 |
| NP | MCF-7 | Breast | 48 | 15.6nM | 3 | F | GSE50705 |
| NP | MCF-7 | Breast | 1 | 1uM | 2 | F | GSE16881 |
| NP | MCF-7 | Breast | 4 | 1uM | 4 | F | GSE16881 |
| NP | MCF-7 | Breast | 48 | 1uM | 2 | F | GSE16881 |
| NP | MCF-7 | Breast | 48 | 1uM | 2 | F | GSE50705 |
| NP | MCF-7 | Breast | 48 | 250nM | 2 | F | GSE50705 |
| NP | MCF-7 | Breast | 48 | 25uM | 1 | F | GSE50705 |
| NP | MCF-7 | Breast | 48 | 3.125uM | 3 | F | GSE50705 |
| NP | MCF-7 | Breast | 48 | 6.25uM | 1 | F | GSE50705 |
| NP | MCF-7 | Breast | 48 | 62.5nM | 2 | F | GSE50705 |
| NP | MCF-7/BUS | Breast | 48 | 10nM | 3 | F | GSE5200 |
| PCB 126 | HepaRG (HPR 116) | Liver | 120 | 100pM | 5 | F | GSE109565 |
| PCB 126 | HepaRG (HPR 116) | Liver | 120 | 10nM | 5 | F | GSE109565 |
| PCB 126 | HepaRG (HPR 116) | Liver | 120 | 1uM | 5 | F | GSE109565 |
| PCB 126 | hMADs | Stem Cell | 48 | 1uM | 4 | N/A | GSE32026 |
| PCB 126 | MCF-7 | Breast | 1 | 10uM | 2 | F | GSE16881 |
| PCB 126 | MCF-7 | Breast | 4 | 10uM | 2 | F | GSE16881 |
| PCB 126 | MCF-7 | Breast | 48 | 10uM | 2 | F | GSE16881 |
| PCB 126 | MCF-7 | Breast | 1 | 2uM | 2 | F | GSE16881 |
| PCB 126 | MCF-7 | Breast | 48 | 2uM | 2 | F | GSE16881 |
| PCB 126 | Primary hepatocyte | Liver | 48 | 100pM | 2 | Mixture | GSE14553 |
| PCB 126 | Primary hepatocyte | Liver | 48 | 10nM | 3 | Mixture | GSE14553 |
| PCB 126 | Primary hepatocyte | Liver | 48 | 10pM | 2 | Mixture | GSE14553 |
| PCB 126 | Primary hepatocyte | Liver | 48 | 10uM | 3 | Mixture | GSE14553 |
| PCB 126 | Primary hepatocyte | Liver | 48 | 1nM | 2 | Mixture | GSE14553 |
| PCB 126 | Primary hepatocyte | Liver | 48 | 1pM | 3 | Mixture | GSE14553 |
| PCB 126 | Primary hepatocyte | Liver | 48 | 1uM | 3 | Mixture | GSE14553 |
| PCB 138 | PBMC | Lymphocyte | 48 | 2.41nM | 3 | F | GSE22632 |
| PCB 153 | HepG2 | Liver | 0.5 | 70uM | 3 | M | GSE6878 |
| PCB 153 | HepG2 | Liver | 1.5 | 70uM | 2 | M | GSE6878 |
| PCB 153 | HepG2 | Liver | 6 | 70uM | 2 | M | GSE6878 |
| PCB 153 | HepG2 | Liver | 18 | 70uM | 3 | M | GSE6878 |
| PCB 153 | HK2 | Kidney | 0.5 | 70uM | 3 | M | GSE23434 |
| PCB 153 | HK2 | Kidney | 6 | 70uM | 3 | M | GSE23434 |
| PCB 153 | HK2 | Kidney | 24 | 70uM | 3 | M | GSE23434 |
| PCB 153 | hMADs | Stem Cell | 48 | 10uM | 4 | N/A | GSE32026 |
| PCB 153 | PBMC | Lymphocyte | 20 | 250uM | 5 | Mixture | GSE19078 |
| PCB 153 | PBMC | Lymphocyte | 20 | 250uM | 5 | Mixture | GSE24891 |
| PCB 153 | PBMC | Lymphocyte | 20 | 25uM | 5 | Mixture | GSE19078 |
| PCB 153 | PBMC | Lymphocyte | 20 | 25uM | 5 | Mixture | GSE24891 |
| PCB 153 | PBMC | Lymphocyte | 48 | 4.63pM | 3 | F | GSE22667 |
| PCB 77 | HepG2 | Liver | 0.5 | 70uM | 3 | M | GSE6878 |
| PCB 77 | HepG2 | Liver | 6 | 70uM | 3 | M | GSE6878 |
| PCB 77 | HepG2 | Liver | 24 | 70uM | 3 | M | GSE6878 |
| PCB 77 | HK2 | Kidney | 0.5 | 40uM | 3 | M | GSE23493 |
| PCB 77 | HK2 | Kidney | 6 | 40uM | 3 | M | GSE23493 |
| PCB 77 | HK2 | Kidney | 24 | 40uM | 3 | M | GSE23493 |
| TCDD | 501Mel | Skin | 48 | 10nM | 2 | F | GSE104869 |
| TCDD | A549 | Lung | 6 | 10nM | 3 | M | GSE109576 |
| TCDD | A549 | Lung | 336 | 10 nM | 6 | M | GSE122733 |
| TCDD | B cell | Lymphocyte | 4 | 30nM | 3 | F | GSE80953 |
| TCDD | B cell | Lymphocyte | 8 | 30nM | 3 | F | GSE80953 |
| TCDD | B cell | Lymphocyte | 24 | 30nM | 3 | F | GSE80953 |
| TCDD | BEAS-2B | Bronchus | 504 | 10nM | 2 | M | GSE83886 |
| TCDD | BEAS-2B | Bronchus | 504 | 2nM | 2 | M | GSE83886 |
| TCDD | CD34+ hemopoietic cells | Lymphocyte | 12 | 20nM | 4 | N/A | GSE24193 |
| TCDD | Fibroblast | Skin | 24 | 1nM | 3 | M | GSE35034 |
| TCDD | HepaRG | Liver | 6 | 100nM | 2 | F | GSE69844 |
| TCDD | HepaRG | Liver | 6 | 10nM | 3 | F | GSE69844* |
| TCDD | HepaRG | Liver | 12 | 10nM | 3 | F | E-MEXP-2458 |
| TCDD | HepaRG | Liver | 24 | 10nM | 3 | F | E-MEXP-2458 |
| TCDD | HepaRG | Liver | 6 | 1nM | 2 | F | GSE69844 |
| TCDD | HepaRG | Liver | 30 | 25nM | 3 | F | GSE46874 |
| TCDD | HepaRG | Liver | 24 | 10 nM | 4 | F | GSE122518 |
| TCDD | HepG2 | Liver | 6 | 100nM | 3 | M | GSE69850 |
| TCDD | HepG2 | Liver | 6 | 10nM | 3 | M | GSE69850* |
| TCDD | HepG2 | Liver | 12 | 10nM | 3 | M | E-MEXP-2458 |
| TCDD | HepG2 | Liver | 12 | 10nM | 3 | M | E-MEXP-2574 |
| TCDD | HepG2 | Liver | 12 | 10nM | 3 | M | E-MEXP-2817 |
| TCDD | HepG2 | Liver | 12 | 10nM | 3 | M | GSE28878 |
| TCDD | HepG2 | Liver | 24 | 10nM | 3 | M | E-MEXP-2458 |
| TCDD | HepG2 | Liver | 24 | 10nM | 3 | M | E-MEXP-2574 |
| TCDD | HepG2 | Liver | 24 | 10nM | 3 | M | E-MEXP-2817 |
| TCDD | HepG2 | Liver | 24 | 10nM | 3 | M | GSE28878 |
| TCDD | HepG2 | Liver | 48 | 10nM | 3 | M | E-MEXP-2574 |
| TCDD | HepG2 | Liver | 48 | 10nM | 3 | M | E-MEXP-2817 |
| TCDD | HepG2 | Liver | 48 | 10nM | 1 | M | GSE22909 |
| TCDD | HepG2 | Liver | 48 | 10nM | 3 | M | GSE28878 |
| TCDD | HepG2 | Liver | 6 | 1nM | 3 | M | GSE69850 |
| TCDD | HL-60 | Blood | 24 | 10nM | 2 | F | GSE16160 |
| TCDD | hMAD | Stem Cell | 48 | 25nM | 4 | - | GSE32026 |
| TCDD | Ishikawa | Endometrium | 6 | 100nM | 3 | F | GSE69849 |
| TCDD | Ishikawa | Endometrium | 6 | 10nM | 3 | F | GSE69849 |
| TCDD | Ishikawa | Endometrium | 6 | 1nM | 3 | F | GSE69849 |
| TCDD | Decidual stromal cells | Decidua | 24 | 10 nM | 3 | F | GSE114552 |
| TCDD | Decidual stromal cells | Decidua | 144 | 10 nM | 2 | F | GSE114552 |
| TCDD | MCF-7 | Breast | 6 | 100nM | 3 | F | GSE69845 |
| TCDD | MCF-7 | Breast | 6 | 100nM | 4 | F | GSE98514 |
| TCDD | MCF-7 | Breast | 1 | 10nM | 2 | F | GSE16881 |
| TCDD | MCF-7 | Breast | 4 | 10nM | 2 | F | GSE16881 |
| TCDD | MCF-7 | Breast | 6 | 10nM | 3 | F | GSE69845 |
| TCDD | MCF-7 | Breast | 6 | 10nM | 4 | F | GSE76608 |
| TCDD | MCF-7 | Breast | 48 | 10nM | 2 | F | GSE16881 |
| TCDD | MCF-7 | Breast | 1 | 1nM | 2 | F | GSE16881 |
| TCDD | MCF-7 | Breast | 4 | 1nM | 2 | F | GSE16881 |
| TCDD | MCF-7 | Breast | 6 | 1nM | 3 | F | GSE69845 |
| TCDD | MCF-7 | Breast | 48 | 1nM | 2 | F | GSE16881 |
| TCDD | Mel1 ES | Stem Cell | 96 | 2nM | 2 | N/A | GSE122002 |
| TCDD | Mesenchymal Stromal cells | Bone Marrow | 24 | 10nM | 5 | N/A | GSE95072 |
| TCDD | NHEK | Neonatal foreskin | 24 | 10nM | 4 | M | GSE36796 |
| TCDD | PBMC | Lymphocyte | 20 | 10uM | 5 | Mixture | GSE19078 |
| TCDD | PBMC | Lymphocyte | 20 | 1uM | 5 | Mixture | GSE19078 |
| TCDD | PMBC | Blood cell | 20 | 10uM | 5 | Mixture | GSE24891 |
| TCDD | PMBC | Blood cell | 20 | 1uM | 5 | Mixture | GSE24891 |
| TCDD | Primary hepatocyte | Liver | 24 | 100fM | 5 | F | GSE34249 |
| TCDD | Primary hepatocyte | Liver | 24 | 100nM | 5 | F | GSE34249 |
| TCDD | Primary hepatocyte | Liver | 24 | 100pM | 5 | F | GSE34249 |
| TCDD | Primary hepatocyte | Liver | 48 | 100pM | 3 | Mixture | GSE14553 |
| TCDD | Primary hepatocyte | Liver | 24 | 10fM | 5 | F | GSE34249 |
| TCDD | Primary hepatocyte | Liver | 48 | 10fM | 3 | Mixture | GSE14553 |
| TCDD | Primary hepatocyte | Liver | 24 | 10nM | 5 | F | GSE34249 |
| TCDD | Primary hepatocyte | Liver | 48 | 10nM | 3 | Mixture | GSE14553 |
| TCDD | Primary hepatocyte | Liver | 24 | 10pM | 5 | F | GSE34249 |
| TCDD | Primary hepatocyte | Liver | 48 | 10pM | 2 | Mixture | GSE14553 |
| TCDD | Primary hepatocyte | Liver | 24 | 1nM | 5 | F | GSE34249 |
| TCDD | Primary hepatocyte | Liver | 48 | 1nM | 2 | Mixture | GSE14553 |
| TCDD | Primary hepatocyte | Liver | 24 | 1pM | 5 | F | GSE34249 |
| TCDD | Primary hepatocyte | Liver | 48 | 1pM | 3 | Mixture | GSE14553 |
| TCDD | Primary hepatocyte | Liver | 24 | 300pM | 5 | F | GSE34249 |
| TCDD | Primary hepatocyte | Liver | 24 | 30pM | 5 | F | GSE34249 |
| TCDD | Primary hepatocyte | Liver | 48 | 316.2nM | 2 | Mixture | GSE14553 |
| TCDD | Primary hepatocyte | Liver | 24 | 3pM | 5 | F | GSE34249 |

Supplemental Table 2. List of thirty searched xenoestrogens.

| **Xenoestrogen** | **Datasets** | **Number** |
| --- | --- | --- |
| Genistein (GEN) | Yes | 97 |
| Bisphenol A (BPA) | Yes | 94 |
| Tetrachlorodibenzo-p-dioxin (TCDD) | Yes | 75 |
| Polychlorinated bisphenol (PCB) | Yes | 36 |
| 17alpha-Ethinylestradiol (EE2) | Yes | 27 |
| 4-Nonylphenol (NP) | Yes | 15 |
| Di-2-ethylhexyl phthalate (DEHP) | Yes | 14 |
| Estrone | Yes | 11 |
| Daidzein | Yes | 10 |
| Diethylstilbestrol (DES) | Yes | 10 |
| Dichlorodiphenyltrichloroethane (DDT) | Yes | 10 |
| Methoxychlor (MOC) | Yes | 10 |
| Atrazine | Yes | 8 |
| Bisphenol S (BPS) | Yes | 5 |
| Bisphenol AF (BPAF) | Yes | 4 |
| Benzophenone-2 | Yes | 1 |
| Zearalenone | Yes | 1 |
| Bisphenol B (BPB) | Yes | 1 |
| Testosterone propionate | No | 0 |
| Triphenylethylene | No | 0 |
| 3-Tetramethylbutyl | No | 0 |
| 4-Cumylphenol | No | 0 |
| 4-Dodecylphenol | No | 0 |
| 5HPP-33 | No | 0 |
| Dodecylphenol | No | 0 |
| Equilin | No | 0 |
| Ethylhexylparaben | No | 0 |
| Meso-hexestrol | No | 0 |
| Mestranol | No | 0 |
| Norgestrel | No | 0 |

Supplemental Table 3. Xenoestrogens with only one available dataset.

| **Chemical** | **Cell Line Type** | **Tissue of origin** | **Time Course (Hr)** | **Dose** | **Replicate #** | **Sex** | **Accession # (*used in analysis)** |
| --- | --- | --- | --- | --- | --- | --- | --- |
| Benzophenone-2 | FTC-238/hrTPO/RSK008 | Thyroid | 48 | 1uM | 4 | M | GSE30961 |
| Bisphenol B | MCF-7 | Breast | 48 | 300nM | 3 | F | GSE85350 |
| Zearalenone | MCF-7 | Breast | 72 | 10nM | 3 | F | GSE15249 |

Supplemental Table 4. Identified Estradiol Gene Expression Arrays Classified by Cell Type, Duration of Exposure, Chemical Concentration, Number of Experimental Replicates, and Genetic Sex of Cell Line.

| **Cell Line Type** | **Tissue of origin** | **Time Course (h)** | **Dose** | **Replicate #** | **Sex** | **Accession # (*used in analysis)** |
| --- | --- | --- | --- | --- | --- | --- |
| 3D epithelial breast culture specimen | Breast | 6 | 10nM | 4 | F | GSE115112 |
| 3D epithelial breast culture specimen | Breast | 24 | 10nM | 4 | F | GSE115112 |
| A549 | Lung | 3 | 1uM | 1 | M | GSE92679 |
| Adipocytes of lean children | Inguinal fat | 24 | 1nM | 5 | M | GSE58516 |
| BEAS-2Bs | Lung | 48 | 10nM | 3 | F | GSE100574 |
| BG-1 | Breast | 18 | 10nM | 3 | F | GSE58324 |
| BLaER1 | Bone Marrow | 3 | 100nM | 2 | F | GSE44700 |
| BLaER1 | Bone Marrow | 6 | 100nM | 2 | F | GSE44700 |
| BLaER1 | Bone Marrow | 9 | 100nM | 2 | F | GSE44700 |
| BLaER1 | Bone Marrow | 12 | 100nM | 2 | F | GSE44700 |
| BLaER1 | Bone Marrow | 18 | 100nM | 2 | F | GSE44700 |
| BLaER1 | Bone Marrow | 24 | 100nM | 2 | F | GSE44700 |
| BLaER1 | Bone Marrow | 36 | 100nM | 2 | F | GSE44700 |
| BLaER1 | Bone Marrow | 48 | 100nM | 2 | F | GSE44700 |
| BLaER1 | Bone Marrow | 72 | 100nM | 2 | F | GSE44700 |
| BLaER1 | Bone Marrow | 120 | 100nM | 2 | F | GSE44700 |
| BLaER1 | Bone Marrow | 168 | 100nM | 2 | F | GSE44700 |
| BT-20 | Breast | 16 | 10 nM | 1 | F | GSE127760 |
| BT-20 | Breast | 16 | 10 nM | 1 | F | GSE127760 |
| BT-20 | Breast | 16 | 10 nM | 1 | F | GSE127760 |
| BT-20 | Breast | 16 | 10 nM | 1 | F | GSE127760 |
| BT474 | Breast | 6 | 1nM | 3 | F | GSE70759 |
| BT-474 | Breast | 2 | N/A | 2 | F | GSE3834 |
| BT-474 | Breast | 4 | N/A | 2 | F | GSE3834 |
| BT-474 | Breast | 8 | N/A | 2 | F | GSE3834 |
| BT-474 | Breast | 12 | N/A | 2 | F | GSE3834 |
| BT-474 | Breast | 16 | 10 nM | 1 | F | GSE127760 |
| BT-474 | Breast | 16 | 10 nM | 1 | F | GSE127760 |
| BT-474 | Breast | 16 | 10 nM | 1 | F | GSE127760 |
| BT-474 | Breast | 16 | 10 nM | 1 | F | GSE127760 |
| BT-474 | Breast | 24 | 1 nM | 2 | F | GSE118942 |
| BT-474 | Breast | 24 | 1 nM | 2 | F | GSE118942 |
| BT-474 | Breast | 24 | 10nM | 2 | F | GSE3529 |
| BT-474 | Breast | 24 | N/A | 2 | F | GSE3834 |
| BT-474 | Breast | N/A | N/A | 2 | F | GSE3529 |
| BT-483 | Breast | 16 | 10 nM | 1 | F | GSE127760 |
| BT-483 | Breast | 16 | 10 nM | 1 | F | GSE127760 |
| BT-483 | Breast | 16 | 10 nM | 1 | F | GSE127760 |
| BT-483 | Breast | 16 | 10 nM | 1 | F | GSE127760 |
| C4-12/Flag.ERβ | Breast | 1 | 10nM | 2 | F | GSE48159 |
| C49R | Breast | 3 | 100nM | 3 | F | GSE104985 |
| C49R | Breast | 6 | 100nM | 3 | F | GSE104985 |
| C70R | Breast | 3 | 100nM | 3 | F | GSE104985 |
| C70R | Breast | 6 | 100nM | 3 | F | GSE104985 |
| CAMA-1 | Breast | 16 | 10 nM | 1 | F | GSE127760 |
| CAMA-1 | Breast | 16 | 10 nM | 1 | F | GSE127760 |
| CAMA-1 | Breast | 16 | 10 nM | 1 | F | GSE127760 |
| CAMA-1 | Breast | 16 | 10 nM | 1 | F | GSE127760 |
| CAMA-1 | Breast | 24 | 1 nM | 2 | F | GSE118942 |
| CAMA-1 | Breast | 24 | 1 nM | 2 | F | GSE118942 |
| Cancer Associated Fibroblasts (Early Stage) | Cervix | 72 | 3.67nM | 4 | F | GSE65227 |
| Cancer Associated Fibroblasts (Late Stage) | Cervix | 72 | 3.67nM | 2 | F | GSE65227 |
| CEBPA-inducible SKH-1 | Bone Marrow | 48 | N/A | 2 | N/A | GSE102730 |
| Dental pulp cells | Dental pulp | 24 | 1nM | 1 | F | GSE44677 |
| Dermal papilla cell | Skin | 24 | 10nM | 3 | F | GSE102949 |
| Eahy 926 | Vascular endothelium | 16 | 10nM | 3 | N/A | GSE72180 |
| Eahy926 | Vascular endothelium | 16 | 10nM | 3 | N/A | GSE51535 |
| EEC | Endometrium | 3 | 100nM | 2 | F | GSE3013 |
| EFM-19 | Breast | 24 | 1 nM | 2 | F | GSE118942 |
| EFM-19 | Breast | 24 | 1 nM | 2 | F | GSE118942 |
| ER:PRL-HeLa | Cervix | 4 | 10nM | 1 | F | GSE22941 |
| H2OS | Bone | 4 | 10nM | 2 | F | GSE1153 |
| H2OS | Bone | 8 | 10nM | 2 | F | GSE1153 |
| H2OS | Bone | 24 | 10nM | 2 | F | GSE1153 |
| H2OS | Bone | 48 | 10nM | 2 | F | GSE1153 |
| H3396 | Breast | 1 | 10nm | 1 | F | GSE32348 |
| H3396 | Breast | 2 | 10nm | 1 | F | GSE32348 |
| H3396 | Breast | 6 | 10nm | 1 | F | GSE32348 |
| H3396 | Breast | 24 | 10nm | 1 | F | GSE32348 |
| H520 | Lung | 3 | 1uM | 1 | M | GSE92679 |
| HBE | Breast | 6 | 10nM | 3 | F | GSE23500 |
| HCC1428 | Breast | 16 | 10 nM | 1 | F | GSE127760 |
| HCC1428 | Breast | 16 | 10 nM | 1 | F | GSE127760 |
| HCC1428 | Breast | 16 | 10 nM | 1 | F | GSE127760 |
| HCC1428 | Breast | 16 | 10 nM | 1 | F | GSE127760 |
| HCC1428 | Breast | N/A | 1nM | 3 | F | GSE75971 |
| HCC1500 | Breast | 24 | 1 nM | 2 | F | GSE118942 |
| HCC1500 | Breast | 24 | 1 nM | 2 |  | GSE118942 |
| HEEC | Endometrium | 6 | 10nM | 3 | F | GSE55691 |
| HepaRG | Liver | 12 | 30uM | 3 | F | E-MEXP-2458 |
| HepaRG | Liver | 12 | 30uM | 3 | M | MEXP-2458* |
| HepaRG | Liver | 48 | 30uM | 2 | F | E-MEXP-2458 |
| HepaRG | Liver | 48 | 30uM | 3 | M | MEXP-2458 |
| HepG2 | Liver | 12 | 30uM | 3 | M | E-MEXP-2458 |
| HepG2 | Liver | 12 | 30uM | 3 | M | E-MEXP-2574 |
| HepG2 | Liver | 12 | 30uM | 3 | M | GSE28878 |
| HepG2 | Liver | 12 | 30uM | 3 | M | MEXP-2458* |
| HepG2 | Liver | 24 | 30uM | 3 | M | E-MEXP-2574 |
| HepG2 | Liver | 24 | 30uM | 3 | M | GSE28878 |
| HepG2 | Liver | 24 | 30uM | 3 | M | GSE51952 |
| HepG2 | Liver | 48 | 1uM | 2 | M | GSE112983 |
| HepG2 | Liver | 48 | 30uM | 3 | M | E-MEXP-2458 |
| HepG2 | Liver | 48 | 30uM | 3 | M | E-MEXP-2574 |
| HepG2 | Liver | 48 | 30uM | 3 | M | GSE28878 |
| HepG2 | Liver | 48 | 30uM | 3 | M | MEXP-2458 |
| HESC | Endometrium | 192 | 10nM | 3 | F | GSE14808 |
| hESF | Endometrium | 6 | 10nM | 4 | F | GSE35287 |
| hESF | Endometrium | 48 | 10nM | 4 | F | GSE35287 |
| hESF | Endometrium | 336 | 10nM | 4 | F | GSE35287 |
| hFOB | Bone | 24 | 10nM | 3 | F | GSE55769 |
| HMC3 | Microglia | N/A | 0.1 nM | 2 | N/A | GSE134782 |
| HMEC | Breast | N/A | 1nM | 1 | F | GSE6548 |
| hSFC | Skin | 24 | 10pM | 3 | - | GSE35034 |
| HT29 | Colon | 24 | 10nM | 2 | F | E-MEXP-3176 |
| HUAECs | Umbilical cord | 24 | 1nM | 5 | F | GSE29881 |
| hUtSMC | Uterus | 6 | 10nM | 3 | F | GSE51470 |
| hUtSMC | Uterus | 24 | 10nM | 5 | F | GSE51470 |
| hUtSMC | Uterus | 72 | 10nM | 3 | F | GSE51470 |
| hUtSMCs | Myometrium | 72 | 10nM | 3 | F | GSE59231 |
| HUVEC | Umbilical cord | 5 | 1uM | 4 | F | GSE1486 |
| HUVEC | Umbilical cord | 5 | 1uM | 4 | F | GSE1486 |
| HUVEC | Umbilical cord | 24 | 1nM | 3 | F | GSE16683 |
| ICI-resistent MCF-7 | Breast | 4 | 10nM | 4 | F | GSE5840 |
| IKK β-MCF-7 | Breast | 72 | N/A | 3 | F | GSE85683 |
| Ishikawa | Endometrium | 3 | 10nM | 1 | F | E-MTAB-822 |
| Ishikawa | Endometrium | 3 | 10nM | 2 | F | GSE107693 |
| Ishikawa | Endometrium | 4 | 10nM | 2 | F | GSE93439 |
| Ishikawa | Uterus | 4 | 10nM | 3 | F | GSE56946 |
| Ishikawa | Endometrium | 8 | 10 nM | 1 | F | GSE129804 |
| Ishikawa | Endometrium | 8 | 10 nM | 2 | F | GSE132428 |
| Ishikawa | Endometrium | 8 | 10 nM | 2 | F | GSE132428 |
| Ishikawa | Endometrium | 8 | 10nM | 2 | F | GSE109892 |
| Ishikawa | Endometrium | 8 | 10nM | 2 | F | GSE38234 |
| Ishikawa | Endometrium | 8 | 10nM | 3 | F | GSE129804 |
| Ishikawa | Endometrium | 12 | 10nM | 1 | F | E-MTAB-822 |
| Ishikawa | Endometrium | 12 | 200pg/mL | 1 | F | GSE31149 |
| Ishikawa | Uterus | 24 | 10nM | 3 | F | GSE56946 |
| Ishikawa | Endometrium | 24 | 10nM | 3 | F | GSE78182 |
| Ishikawa | Endometrium | 24 | 10nM | 10 | F | GSE3762 |
| Ishikawa | Endometrium | 24 | 10nM | 12 | F | GSE3762 |
| Ishikawa | Endometrium | 24 | 200pg/mL | 3 | F | GSE31149 |
| Ishikawa | Endometrium | 24 | 30nM | 2 | F | GSE115608 |
| Ishikawa | Endometrium | 24 | 3nM | 2 | F | GSE115608 |
| Ishikawa | Endometrium | 48 | 10nM | 12 | F | GSE3762 |
| Ishikawa | Endometrium | 48 | 10nM | 14 | F | GSE3762 |
| Ishikawa | Endometrium | 72 | 1nM | 3 | F | GSE68333 |
| Ishikawa | Endometrium | 240 | 10 nM | 2 | F | GSE132428 |
| Ishikawa | Endometrium | 240 | 10 nM | 2 | F | GSE132428 |
| Ishikawa | Endometrium | 360 | 10 nM | 2 | F | GSE132428 |
| Ishikawa | Endometrium | 360 | 10 nM | 2 | F | GSE132428 |
| Ishikawa | Endometrium | 480 | 10 nM | 2 | F | GSE132428 |
| Ishikawa | Endometrium | 480 | 10 nM | 2 | F | GSE132428 |
| Ishikawa | Endometrium | 600 | 10 nM | 2 | F | GSE132428 |
| Ishikawa | Endometrium | 600 | 10 nM | 2 | F | GSE132428 |
| JHUEM14 | Endometrium | 3 | 10nM | 2 | F | GSE107693 |
| K562 p42-C/EBPa-ER expressing cells | Bone Marrow | 6 | 1uM | 4 | F | GSE43998 |
| LNCaP | Prostate | 1 | 50nM | 1 | M | GSE37531 |
| LNCaP | Prostate | 24 | 100nM | 1 | M | GSE58615 |
| LNCaP | Prostate | 24 | 50nM | 1 | M | GSE37531 |
| Mature luminal cells | Breast | 24 | 10 nM | 7 | F | GSE99680 |
| Mature luminal cells | Breast | 24 | 10 nM | 7 | F | GSE99680 |
| MCF-7 | Breast | 0.0833 | 10nM | 1 | F | GSE62789 |
| MCF-7 | Breast | 0.1667 | 100nM | 2 | F | GSE43835 |
| MCF-7 | Breast | 0.1667 | 100nM | 3 | F | GSE41324 |
| MCF-7 | Breast | 0.1667 | 10nM | 1 | F | GSE62789 |
| MCF-7 | Breast | 0.333 | 10nM | 1 | F | GSE62789 |
| MCF-7 | Breast | 0.41667 | 100nM | 2 | F | GSE41324 |
| MCF-7 | Breast | 0.667 | 100nM | 2 | F | GSE43835 |
| MCF-7 | Breast | 0.667 | 100nM | 3 | F | GSE41324 |
| MCF-7 | Breast | 0.667 | 100nM | 3 | F | GSE59531 |
| MCF-7 | Breast | 0.667 | 10nM | 1 | F | GSE62789 |
| MCF-7 | Breast | 0.75 | 10nM | 2 | F | GSE86316 |
| MCF-7 | Breast | 1 | 100nM | 2 | F | GSE16881 |
| MCF-7 | Breast | 1 | 100nM | 2 | F | GSE45822 |
| MCF-7 | Breast | 1 | 100nM | 2 | F | GSE60271 |
| MCF-7 | Breast | 1 | 100nM | 2 | F | GSE62228 |
| MCF-7 | Breast | 1 | 10nM | 2 | F | E-TABM-742 |
| MCF-7 | Breast | 1 | 10nM | 2 | F | GSE16881 |
| MCF-7 | Breast | 1 | 10nM | 3 | F | GSE18592 |
| MCF-7 | Breast | 1 | 10nM | 3 | F | GSE21618 |
| MCF-7 | Breast | 1 | 10nM | 3 | F | GSE78167 |
| MCF-7 | Breast | 1 | 10nM | 4 | F | GSE22012 |
| MCF-7 | Breast | 1 | 1nM | 2 | F | GSE16881 |
| MCF-7 | Breast | 1 | N/A | 1 | F | GSE3834 |
| MCF-7 | Breast | 1.333 | 10nM | 1 | F | GSE62789 |
| MCF-7 | Breast | 2 | 0.01nM | 3 | F | GSE84981 |
| MCF-7 | Breast | 2 | 0.1nM | 3 | F | GSE84981 |
| MCF-7 | Breast | 2 | 10nM | 2 | F | E-TABM-742 |
| MCF-7 | Breast | 2 | 10nM | 2 | F | GSE108308 |
| MCF-7 | Breast | 2 | 10nM | 2 | F | GSE55922 |
| MCF-7 | Breast | 2 | 10nM | 3 | F | GSE11467 |
| MCF-7 | Breast | 2 | 10nM | 3 | F | GSE21618 |
| MCF-7 | Breast | 2 | 10nM | 3 | F | GSE64590 |
| MCF-7 | Breast | 2 | 10nM | 3 | F | GSE78167 |
| MCF-7 | Breast | 2 | 1nM | 3 | F | GSE84981 |
| MCF-7 | Breast | 2 | N/A | 2 | F | GSE3834 |
| MCF-7 | Breast | 2.667 | 100nM | 1 | F | GSE43070 |
| MCF-7 | Breast | 2.667 | 10nM | 1 | F | GSE62789 |
| MCF-7 | Breast | 3 | 100 nM | 2 | F | GSE109570 |
| MCF-7 | Breast | 3 | 100nM | 1 | F | GSE85088 |
| MCF-7 | Breast | 3 | 100nM | 3 | F | GSE104985 |
| MCF-7 | Breast | 3 | 100nM | 3 | F | GSE13458 |
| MCF-7 | Breast | 3 | 100nM | 3 | F | GSE59908 |
| MCF-7 | Breast | 3 | 100pM | 2 | F | GSE89700 |
| MCF-7 | Breast | 3 | 100pM | 2 | F | GSE89700 |
| MCF-7 | Breast | 3 | 100pM | 3 | F | GSE60759 |
| MCF-7 | Breast | 3 | 10nM | 1 | F | E-MTAB-822 |
| MCF-7 | Breast | 3 | 10nM | 2 | F | GSE107693 |
| MCF-7 | Breast | 3 | 10nM | 2 | F | GSE28006 |
| MCF-7 | Breast | 3 | 10nM | 2 | F | GSE59345 |
| MCF-7 | Breast | 3 | 10nM | 2 | F | GSE76507 |
| MCF-7 | Breast | 3 | 10nM | 3 | F | GSE21618 |
| MCF-7 | Breast | 3 | 10nM | 3 | F | GSE26834 |
| MCF-7 | Breast | 3 | 10nM | 3 | F | GSE78167 |
| MCF-7 | Breast | 3 | 10nM | 4 | F | GSE67295 |
| MCF-7 | Breast | 3 | 10uM | 3 | F | GSE76453 |
| MCF-7 | Breast | 3 | 1nM | 3 | F | GSE11506 |
| MCF-7 | Breast | 3 | 1nM | 5 | F | GSE48927 |
| MCF-7 | Breast | 3 | 1uM | 1 | F | GSE32667 |
| MCF-7 | Breast | 3 | 367.134nM | 2 | F | GSE39417 |
| MCF-7 | Breast | 3 | N/A | 12 | F | GSE107858 |
| MCF-7 | Breast | 4 | 0.01nM | 3 | F | GSE84981 |
| MCF-7 | Breast | 4 | 0.1nM | 3 | F | GSE84981 |
| MCF-7 | Breast | 4 | 10 nM | 2 | F | GSE135427 |
| MCF-7 | Breast | 4 | 10 nM | 4 | F | GSE107924 |
| MCF-7 | Breast | 4 | 10 nM | 4 | F | GSE107924 |
| MCF-7 | Breast | 4 | 100 nM | 1 | F | GSE137559 |
| MCF-7 | Breast | 4 | 100nM | 2 | F | GSE16881 |
| MCF-7 | Breast | 4 | 100nM | 3 | F | GSE10466 |
| MCF-7 | Breast | 4 | 10nM | 1 | F | E-MTAB-4923 |
| MCF-7 | Breast | 4 | 10nM | 1 | F | GSE2987 |
| MCF-7 | Breast | 4 | 10nM | 1 | F | GSE2987 |
| MCF-7 | Breast | 4 | 10nM | 1 | F | GSE39786 |
| MCF-7 | Breast | 4 | 10nM | 2 | F | E-SMDB-1443 |
| MCF-7 | Breast | 4 | 10nM | 2 | F | E-TABM-742 |
| MCF-7 | Breast | 4 | 10nM | 2 | F | GSE120756 |
| MCF-7 | Breast | 4 | 10nM | 2 | F | GSE16881 |
| MCF-7 | Breast | 4 | 10nM | 2 | F | GSE73663 |
| MCF-7 | Breast | 4 | 10nM | 3 | F | GSE15717 |
| MCF-7 | Breast | 4 | 10nM | 3 | F | GSE24592 |
| MCF-7 | Breast | 4 | 10nM | 3 | F | GSE68918 |
| MCF-7 | Breast | 4 | 10nM | 3 | F | GSE78167 |
| MCF-7 | Breast | 4 | 10nM | 3 | F | GSE79761 |
| MCF-7 | Breast | 4 | 10nM | 4 | F | GSE10618 |
| MCF-7 | Breast | 4 | 10nM | 4 | F | GSE5840 |
| MCF-7 | Breast | 4 | 1nM | 2 | F | GSE16881 |
| MCF-7 | Breast | 4 | 1nM | 3 | F | GSE57935 |
| MCF-7 | Breast | 4 | 1nM | 3 | F | GSE84981 |
| MCF-7 | Breast | 4 | 6nM | 3 | F | GSE9936 |
| MCF-7 | Breast | 4 | N/A | 2 | F | GSE3834 |
| MCF-7 | Breast | 4 | N/A | 3 | F | GSE23445 |
| MCF-7 | Breast | 5 | 10nM | 3 | F | GSE78167 |
| MCF-7 | Breast | 5.333 | 10nM | 1 | F | GSE62789 |
| MCF-7 | Breast | 6 | 10 nM | 3 | F | GSE124270 |
| MCF-7 | Breast | 6 | 100 nM | 2 | F | GSE125606 |
| MCF-7 | Breast | 6 | 100 nM | 2 | F | GSE125606 |
| MCF-7 | Breast | 6 | 100nM | 2 | F | GSE5258 |
| MCF-7 | Breast | 6 | 100nM | 3 | F | GSE104985 |
| MCF-7 | Breast | 6 | 100nM | 3 | F | GSE23610 |
| MCF-7 | Breast | 6 | 100nM | 3 | F | GSE45643 |
| MCF-7 | Breast | 6 | 100nM | 3 | F | GSE59908 |
| MCF-7 | Breast | 6 | 100nM | 6 | F | GSE39623 |
| MCF-7 | Breast | 6 | 10nM | 1 | F | E-TABM-1194 |
| MCF-7 | Breast | 6 | 10nM | 2 | F | E-TABM-742 |
| MCF-7 | Breast | 6 | 10nM | 2 | F | GSE46856 |
| MCF-7 | Breast | 6 | 10nM | 3 | F | GSE20081 |
| MCF-7 | Breast | 6 | 10nM | 3 | F | GSE21618 |
| MCF-7 | Breast | 6 | 10nM | 3 | F | GSE30931 |
| MCF-7 | Breast | 6 | 10nM | 3 | F | GSE38252 |
| MCF-7 | Breast | 6 | 10nM | 3 | F | GSE78167 |
| MCF-7 | Breast | 6 | 10nM | 4 | F | GSE20342 |
| MCF-7 | Breast | 6 | 10nM | 4 | F | GSE36683 |
| MCF-7 | Breast | 6 | 10nM | 6 | F | E-MTAB-2577 |
| MCF-7 | Breast | 6 | 10nM | 8 | F | GSE5258 |
| MCF-7 | Breast | 6 | 1nM | 1 | F | GSE71297 |
| MCF-7 | Breast | 6 | 1nM | 3 | F | GSE11506 |
| MCF-7 | Breast | 6 | 1nM | 3 | F | GSE70759 |
| MCF-7 | Breast | 6 | 1nM | 4 | F | GSE74146 |
| MCF-7 | Breast | 6 | 1nM | 5 | F | GSE48927 |
| MCF-7 | Breast | 6 | 1nM | 6 | F | E-MTAB-788 |
| MCF-7 | Breast | 6 | 1nM | 6 | F | GSE48924 |
| MCF-7 | Breast | 6 | 1nM | 6 | F | GSE48925 |
| MCF-7 | Breast | 6 | N/A | 12 | F | GSE107858 |
| MCF-7 | Breast | 8 | 0.01nM | 3 | F | GSE84981 |
| MCF-7 | Breast | 8 | 0.1nM | 3 | F | GSE84981 |
| MCF-7 | Breast | 8 | 100pM | 6 | F | GSE77244 |
| MCF-7 | Breast | 8 | 10nM | 1 | F | GSE2987 |
| MCF-7 | Breast | 8 | 10nM | 1 | F | GSE2987 |
| MCF-7 | Breast | 8 | 10nM | 2 | F | E-SMDB-1443 |
| MCF-7 | Breast | 8 | 10nM | 2 | F | E-TABM-742 |
| MCF-7 | Breast | 8 | 10nM | 2 | F | GSE848 |
| MCF-7 | Breast | 8 | 10nM | 2 | F | GSE848 |
| MCF-7 | Breast | 8 | 10nM | 3 | F | GSE78167 |
| MCF-7 | Breast | 8 | 10nM | 3 | F | GSE78285 |
| MCF-7 | Breast | 8 | 10pM | 3 | F | GSE77244 |
| MCF-7 | Breast | 8 | 19nM | 3 | F | GSE15548 |
| MCF-7 | Breast | 8 | 1nM | 3 | F | GSE84981 |
| MCF-7 | Breast | 8 | 1nM | 8 | F | GSE77244 |
| MCF-7 | Breast | 8 | N/A | 2 | F | GSE3834 |
| MCF-7 | Breast | 10 | 100nM | 3 | F | GSE24065 |
| MCF-7 | Breast | 10 | 1nM | 3 | F | GSE24065 |
| MCF-7 | Breast | 10.667 | 10nM | 1 | F | GSE62789 |
| MCF-7 | Breast | 12 | 10 nM | 1 | F | GSE133423 |
| MCF-7 | Breast | 12 | 10 nM | 2 | F | GSE132433 |
| MCF-7 | Breast | 12 | 100nM | 3 | F | GSE22213 |
| MCF-7 | Breast | 12 | 10nM | 1 | F | E-MTAB-822 |
| MCF-7 | Breast | 12 | 10nM | 1 | F | E-TABM-1194 |
| MCF-7 | Breast | 12 | 10nM | 1 | F | GSE26740 |
| MCF-7 | Breast | 12 | 10nM | 1 | F | GSE80366 |
| MCF-7 | Breast | 12 | 10nM | 1 | F | GSE93193 |
| MCF-7 | Breast | 12 | 10nM | 2 | F | E-MTAB-1196 |
| MCF-7 | Breast | 12 | 10nM | 2 | F | E-TABM-742 |
| MCF-7 | Breast | 12 | 10nM | 3 | F | GSE11266 |
| MCF-7 | Breast | 12 | 10nM | 3 | F | GSE11352 |
| MCF-7 | Breast | 12 | 10nM | 3 | F | GSE21618 |
| MCF-7 | Breast | 12 | 10nM | 3 | F | GSE78167 |
| MCF-7 | Breast | 12 | 10nM | 4 | F | GSE10618 |
| MCF-7 | Breast | 12 | 1nM | 5 | F | GSE48927 |
| MCF-7 | Breast | 12 | N/A | 2 | F | GSE3834 |
| MCF-7 | Breast | 12 | N/A | 10 | F | GSE107858 |
| MCF-7 | Breast | 12 | N/A | 20 | F | GSE119455 |
| MCF-7 | Breast | 16 | 10 nM | 1 | F | GSE127760 |
| MCF-7 | Breast | 16 | 10 nM | 1 | F | GSE127760 |
| MCF-7 | Breast | 16 | 10 nM | 1 | F | GSE127760 |
| MCF-7 | Breast | 16 | 10 nM | 1 | F | GSE127760 |
| MCF-7 | Breast | 16 | 10nM | 1 | F | GSE101860 |
| MCF-7 | Breast | 16 | 10nM | 2 | F | E-TABM-742 |
| MCF-7 | Breast | 16 | 5nM | 3 | F | GSE108883 |
| MCF-7 | Breast | 18 | 10nM | 3 | F | GSE58324 |
| MCF-7 | Breast | 20 | 10nM | 2 | F | E-TABM-742 |
| MCF-7 | Breast | 21.33 | 10nM | 1 | F | GSE62789 |
| MCF-7 | Breast | 24 | 0.01nM | 3 | F | GSE84981 |
| MCF-7 | Breast | 24 | 0.1nM | 3 | F | GSE84981 |
| MCF-7 | Breast | 24 | 1 nM | 2 | F | GSE118942 |
| MCF-7 | Breast | 24 | 1 nM | 2 | F | GSE118942 |
| MCF-7 | Breast | 24 | 1 nM | 4 | F | GSE136595 |
| MCF-7 | Breast | 24 | 1 nM | 4 | F | GSE136595 |
| MCF-7 | Breast | 24 | 10nM | 1 | F | E-MTAB-4923 |
| MCF-7 | Breast | 24 | 10nM | 1 | F | E-TABM-1194 |
| MCF-7 | Breast | 24 | 10nM | 1 | F | GSE2987 |
| MCF-7 | Breast | 24 | 10nM | 1 | F | GSE2987 |
| MCF-7 | Breast | 24 | 10nM | 1 | F | GSE38621 |
| MCF-7 | Breast | 24 | 10nM | 2 | F | E-SMDB-1443 |
| MCF-7 | Breast | 24 | 10nM | 2 | F | E-TABM-742 |
| MCF-7 | Breast | 24 | 10nM | 2 | F | GSE24592 |
| MCF-7 | Breast | 24 | 10nM | 2 | F | GSE3529 |
| MCF-7 | Breast | 24 | 10nM | 2 | F | GSE4006 |
| MCF-7 | Breast | 24 | 10nM | 2 | F | GSE42347 |
| MCF-7 | Breast | 24 | 10nM | 2 | F | GSE45047 |
| MCF-7 | Breast | 24 | 10nM | 2 | F | GSE45557 |
| MCF-7 | Breast | 24 | 10nM | 2 | F | GSE48989 |
| MCF-7 | Breast | 24 | 10nM | 2 | F | GSE53394 |
| MCF-7 | Breast | 24 | 10nM | 2 | F | GSE56066 |
| MCF-7 | Breast | 24 | 10nM | 2 | F | GSE56245 |
| MCF-7 | Breast | 24 | 10nM | 2 | F | GSE73663 |
| MCF-7 | Breast | 24 | 10nM | 2 | F | GSE8383 |
| MCF-7 | Breast | 24 | 10nM | 2 | F | GSE99626 |
| MCF-7 | Breast | 24 | 10nM | 3 | F | GSE102367 |
| MCF-7 | Breast | 24 | 10nM | 3 | F | GSE11352 |
| MCF-7 | Breast | 24 | 10nM | 3 | F | GSE26834 |
| MCF-7 | Breast | 24 | 10nM | 3 | F | GSE31118 |
| MCF-7 | Breast | 24 | 10nM | 3 | F | GSE36586 |
| MCF-7 | Breast | 24 | 10nM | 3 | F | GSE4025 |
| MCF-7 | Breast | 24 | 10nM | 3 | F | GSE42619 |
| MCF-7 | Breast | 24 | 10nM | 3 | F | GSE43702 |
| MCF-7 | Breast | 24 | 10nM | 3 | F | GSE78167 |
| MCF-7 | Breast | 24 | 10nM | 4 | F | GSE10618 |
| MCF-7 | Breast | 24 | 10nM | 4 | F | GSE20342 |
| MCF-7 | Breast | 24 | 10nM | 4 | F | GSE21618 |
| MCF-7 | Breast | 24 | 10nM | 7 | F | GSE51403 |
| MCF-7 | Breast | 24 | 1nm | 1 | F | GSE59536 |
| MCF-7 | Breast | 24 | 1nM | 2 | F | GSE27375 |
| MCF-7 | Breast | 24 | 1nM | 2 | F | GSE6800 |
| MCF-7 | Breast | 24 | 1nM | 3 | F | GSE46924 |
| MCF-7 | Breast | 24 | 1nM | 3 | F | GSE57935 |
| MCF-7 | Breast | 24 | 1nM | 3 | F | GSE84981 |
| MCF-7 | Breast | 24 | 1nM | 4 | F | GSE89888 |
| MCF-7 | Breast | 24 | 1nM | 5 | F | GSE48924 |
| MCF-7 | Breast | 24 | 1nM | 5 | F | GSE48927 |
| MCF-7 | Breast | 24 | 1nM | 9 | F | GSE48925 |
| MCF-7 | Breast | 24 | 1nM | 10 | F | GSE35428 |
| MCF-7 | Breast | 24 | 25nM | 4 | F | GSE8597 |
| MCF-7 | Breast | 24 | 6nM | 5 | F | GSE9936 |
| MCF-7 | Breast | 24 | N/A | 2 | F | GSE112243 |
| MCF-7 | Breast | 24 | N/A | 2 | F | GSE3834 |
| MCF-7 | Breast | 24 | N/A | 2 | F | GSE6803 |
| MCF-7 | Breast | 28 | 10nM | 2 | F | E-TABM-742 |
| MCF-7 | Breast | 32 | 10nM | 2 | F | E-TABM-742 |
| MCF-7 | Breast | 48 | 100fM | 5 | F | GSE50705 |
| MCF-7 | Breast | 48 | 100nM | 2 | F | GSE16881 |
| MCF-7 | Breast | 48 | 100nM | 2 | F | GSE50705 |
| MCF-7 | Breast | 48 | 100pM | 3 | F | GSE85350 |
| MCF-7 | Breast | 48 | 100pM | 3 | F | GSE85350 |
| MCF-7 | Breast | 48 | 100pM | 4 | F | GSE50705 |
| MCF-7 | Breast | 48 | 10nM | 2 | F | GSE16881 |
| MCF-7 | Breast | 48 | 10nM | 2 | F | GSE848 |
| MCF-7 | Breast | 48 | 10nM | 2 | F | GSE848 |
| MCF-7 | Breast | 48 | 10nM | 3 | F | GSE11352 |
| MCF-7 | Breast | 48 | 10nM | 3 | F | GSE17460 |
| MCF-7 | Breast | 48 | 10nM | 3 | F | GSE21618 |
| MCF-7 | Breast | 48 | 10nM | 3 | F | GSE50705 |
| MCF-7 | Breast | 48 | 10nM | 3 | F | GSE65620 |
| MCF-7 | Breast | 48 | 10pM | 3 | F | GSE85350 |
| MCF-7 | Breast | 48 | 10pM | 3 | F | GSE85350 |
| MCF-7 | Breast | 48 | 10pM | 5 | F | GSE50705 |
| MCF-7 | Breast | 48 | 1nM | 2 | F | GSE16881 |
| MCF-7 | Breast | 48 | 1nM | 3 | F | GSE50705 |
| MCF-7 | Breast | 48 | 1nM | 3 | F | GSE85350 |
| MCF-7 | Breast | 48 | 1pM | 3 | F | GSE85350 |
| MCF-7 | Breast | 48 | 1pM | 5 | F | GSE50705 |
| MCF-7 | Breast | 48 | 1uM | 3 | F | GSE50705 |
| MCF-7 | Breast | 48 | 20pM | 5 | F | GSE50705 |
| MCF-7 | Breast | 48 | 2pM | 4 | F | GSE50705 |
| MCF-7 | Breast | 48 | 40pM | 5 | F | GSE50705 |
| MCF-7 | Breast | 48 | 4pM | 5 | F | GSE50705 |
| MCF-7 | Breast | 48 | 500fM | 5 | F | GSE50705 |
| MCF-7 | Breast | 48 | 50fM | 5 | F | GSE50705 |
| MCF-7 | Breast | 48 | 60pM | 5 | F | GSE50705 |
| MCF-7 | Breast | 48 | 6pM | 5 | F | GSE50705 |
| MCF-7 | Breast | 48 | 80pM | 8 | F | GSE50705 |
| MCF-7 | Breast | 48 | 8pM | 3 | F | GSE50705 |
| MCF-7 | Breast | 48 | N/A | 3 | F | GSE87701 |
| MCF-7 | Breast | 72 | 1 nM | 3 | F | GSE120931 |
| MCF-7 | Breast | 72 | 1 nM | 3 | F | GSE120931 |
| MCF-7 | Breast | 72 | 10nM | 1 | F | E-TABM-1194 |
| MCF-7 | Breast | 72 | 10nM | 2 | F | GSE15249 |
| MCF-7 | Breast | 72 | 10nM | 3 | F | GSE108549 |
| MCF-7 | Breast | 72 | 10nM | 3 | F | GSE64155 |
| MCF-7 | Breast | 72 | 10nM | 3 | F | GSE86565 |
| MCF-7 | Breast | 72 | 10nM | 3 | F | GSE86565 |
| MCF-7 | Breast | 72 | 10nM | 6 | F | E-MTAB-2729 |
| MCF-7 | Breast | 168 | 1nM | 3 | F | E-MTAB-922 |
| MCF-7 | Breast | 336 | 1nM | 3 | F | E-MTAB-922 |
| MCF-7 | Breast | 504 | 1nM | 3 | F | E-MTAB-922 |
| MCF-7 | Breast | >5040 | 1pM | 3 | F | GSE33287 |
| MCF-7 | Breast | >5040 | 50pM | 3 | F | GSE33287 |
| MCF-7 | Breast | N/A | 10nM | 2 | F | GSE12291 |
| MCF-7 | Breast | N/A | 10nM | 3 | F | E-MTAB-6893 |
| MCF-7 | Breast | N/A | 1nM | 3 | F | GSE100075 |
| MCF-7 | Breast | N/A | 1nM | 3 | F | GSE75971 |
| MCF-7 | Breast | N/A | N/A | 2 | F | GSE15805 |
| MCF-7 | Breast | N/A | N/A | 2 | F | GSE3529 |
| MCF-7 | Breast | N/A | N/A | 3 | F | GSE94493 |
| MCF7 C-TAP-ER-alpha | Breast | 12 | 10nM | 2 | F | E-MTAB-1196 |
| MCF-7/BUS | Breast | 48 | 100pM | 5 | F | GSE4668 |
| MCF-7/BUS | Breast | 48 | 10pM | 5 | F | GSE4668 |
| MCF-7/BUS | Breast | 48 | 30pM | 3 | F | GSE5200 |
| MCF-7/BUS | Breast | 48 | 30pM | 5 | F | GSE4668 |
| MCF-7/BUS | Breast | 48 | 60pM | 5 | F | GSE4668 |
| MCF7/Rx2dox | Breast | 48 | 10nM | 2 | F | GSE30597 |
| MCF-7/Rx2dox | Breast | 48 | 10nM | 3 | F | GSE65616 |
| MCF-7:2A | Breast | 2 | 1nM | 6 | F | GSE29917 |
| MCF-7:2A | Breast | 6 | 1nM | 6 | F | GSE29917 |
| MCF-7:2A | Breast | 12 | 1nM | 6 | F | GSE29917 |
| MCF-7:2A | Breast | 24 | 1nM | 6 | F | GSE29917 |
| MCF-7:2A | Breast | 48 | 1nM | 6 | F | GSE29917 |
| MCF-7:2A | Breast | 72 | 1nM | 6 | F | GSE29917 |
| MCF-7:2A | Breast | 96 | 1nM | 6 | F | GSE29917 |
| MCF-7:5C | Breast | 2 | 1nM | 6 | F | GSE29917 |
| MCF-7:5C | Breast | 6 | 1nM | 6 | F | GSE29917 |
| MCF-7:5C | Breast | 12 | 1nM | 6 | F | GSE29917 |
| MCF-7:5C | Breast | 24 | 1nM | 6 | F | GSE29917 |
| MCF-7:5C | Breast | 48 | 1nM | 6 | F | GSE29917 |
| MCF-7:5C | Breast | 72 | 1nM | 1 | F | GSE59535 |
| MCF-7:5C | Breast | 72 | 1nM | 6 | F | GSE29917 |
| MCF-7:5C | Breast | 96 | 1nM | 6 | F | GSE29917 |
| MCF-7:PF | Breast | 168 | 1nM | 3 | F | GSE54171 |
| MCF-7:WS8 | Breast | 2 | 1nM | 6 | F | GSE29917 |
| MCF-7:WS8 | Breast | 6 | 1nM | 6 | F | GSE29917 |
| MCF-7:WS8 | Breast | 12 | 1nM | 6 | F | GSE29917 |
| MCF-7:WS8 | Breast | 24 | 1nM | 6 | F | GSE29917 |
| MCF-7:WS8 | Breast | 48 | 1nM | 6 | F | GSE29917 |
| MCF-7:WS8 | Breast | 72 | 1nM | 6 | F | GSE29917 |
| MCF-7:WS8 | Breast | 96 | 1nM | 6 | F | GSE29917 |
| MCF-7aro | Breast | 48 | 1nM | 3 | F | GSE114260 |
| MCF-7aro | Breast | 168 | 1nM | 3 | F | GSE2225 |
| MCF-7aro | Breast | 168 | 1nM | 3 | F | GSE2225 |
| MCF7-B7TamS | Breast | 4 | 10nM | 3 | F | GSE26459 |
| MCF7-G11TamR | Breast | 4 | 10nM | 3 | F | GSE26459 |
| MCF7-tet-on-CARM1 | Breast | 4 | 10nM | 3 | F | GSE26259 |
| MCF7-tet-on-CARM1 | Breast | 4 | 10nM | 3 | F | GSE26454 |
| MCF7-tet-on-shCtr9 | Breast | 4 | 10nM | 3 | F | GSE73388 |
| MDA MB 134VI | Breast | 3 | 1nM | 4 | F | GSE50693 |
| MDA MB 134VI | Breast | 24 | 1nM | 4 | F | GSE50693 |
| MDA231-derived brain trophic 231BR cells | Brain | 24 | 10nM | 3 | F | GSE71272 |
| MDA-MB-134 | Breast | 16 | 10 nM | 1 | F | GSE127760 |
| MDA-MB-134 | Breast | 16 | 10 nM | 1 | F | GSE127760 |
| MDA-MB-134 | Breast | 16 | 10 nM | 1 | F | GSE127760 |
| MDA-MB-134 | Breast | 16 | 10 nM | 1 | F | GSE127760 |
| MDA-MB-134 | Breast | 24 | 1 nM | 2 | F | GSE128911 |
| MDA-MB-134 | Breast | 24 | 1 nM | 2 | F | GSE128911 |
| MDA-MB-157 | Breast | 16 | 10 nM | 1 | F | GSE127760 |
| MDA-MB-157 | Breast | 16 | 10 nM | 1 | F | GSE127760 |
| MDA-MB-157 | Breast | 16 | 10 nM | 1 | F | GSE127760 |
| MDA-MB-157 | Breast | 16 | 10 nM | 1 | F | GSE127760 |
| MDA-MB-175 | Breast | 16 | 10 nM | 1 | F | GSE127760 |
| MDA-MB-175 | Breast | 16 | 10 nM | 1 | F | GSE127760 |
| MDA-MB-175 | Breast | 16 | 10 nM | 1 | F | GSE127760 |
| MDA-MB-175 | Breast | 16 | 10 nM | 1 | F | GSE127760 |
| MDA-MB-231 | Breast | 1 | 10nM | 2 | F | GSE1045 |
| MDA-MB-231 | Breast | 2 | 10nM | 2 | F | GSE1045 |
| MDA-MB-231 | Breast | 3 | 1uM | 1 | F | GSE32668 |
| MDA-MB-231 | Breast | 3 | 1uM | 1 | F | GSE60879 |
| MDA-MB-231 | Breast | 6 | 1nM | 6 | F | GSE9757 |
| MDA-MB-231 | Breast | 6 | 1nM | 6 | F | GSE9758 |
| MDA-MB-231 | Breast | 6 | 1nM | 6 | F | GSE9759 |
| MDA-MB-231 | Breast | 48 | 10nM | 3 | F | GSE2251 |
| MDA-MB-231 | Breast | 120 | 1nM | 3 | F | GSE108980 |
| MDA-MB-231 | Breast | 0.333 | 100nM | 4 | F | GSE95122 |
| MDA-MB-231 | Breast | 0.75 | 100nM | 4 | F | GSE95122 |
| MDA-MB-231 | Breast | 1 | 10nM | 2 | F | GSE1045 |
| MDA-MB-231 | Breast | 1 | 10nM | 2 | F | GSE22593 |
| MDA-MB-231 | Breast | 2 | 10nM | 2 | F | GSE1045 |
| MDA-MB-231 | Breast | 2 | 10nM | 2 | F | GSE22593 |
| MDA-MB-231 | Breast | 3 | 1uM | 1 | F | GSE39718 |
| MDA-MB-231 | Breast | 4 | 10nM | 2 | F | GSE22593 |
| MDA-MB-231 | Breast | 6 | 1nM | 6 | F | GSE9757 |
| MDA-MB-231 | Breast | 6 | 1nM | 6 | F | GSE9758 |
| MDA-MB-231 | Breast | 6 | 1nM | 6 | F | GSE9759 |
| MDA-MB-231 | Breast | 8 | 10nM | 2 | F | GSE22593 |
| MDA-MB-231 | Breast | 24 | 10nM | 2 | F | GSE22593 |
| MDA-MB-231 | Breast | 24 | 10nM | 3 | F | GSE30574 |
| MDA-MB-231 | Breast | 48 | 10nM | 3 | F | GSE2251 |
| MDA-MB-231 | Breast | 216 | 10nM | 3 | F | GSE30574 |
| MDA-MB-330 | Breast | 24 | 1 nM | 2 | F | GSE118942 |
| MDA-MB-330 | Breast | 24 | 1 nM | 2 | F | GSE118942 |
| MDA-MB-361 | Breast | 16 | 10 nM | 1 | F | GSE127760 |
| MDA-MB-361 | Breast | 16 | 10 nM | 1 | F | GSE127760 |
| MDA-MB-361 | Breast | 16 | 10 nM | 1 | F | GSE127760 |
| MDA-MB-361 | Breast | 16 | 10 nM | 1 | F | GSE127760 |
| MDA-MB-415 | Breast | 16 | 10 nM | 1 | F | GSE127760 |
| MDA-MB-415 | Breast | 16 | 10 nM | 1 | F | GSE127760 |
| MDA-MB-415 | Breast | 16 | 10 nM | 1 | F | GSE127760 |
| MDA-MB-415 | Breast | 16 | 10 nM | 1 | F | GSE127760 |
| MDA-MB-468 | Breast | 4 | 10nM | 3 | F | GSE71960 |
| NCI-H2196 | Lung | N/A | 10nM | 1 | M | E-MTAB-2706 |
| NCI-H2198 | Lung | N/A | 10nM | 1 | M | E-MTAB-2706 |
| OCI-Ly7 | B-cell lymphoma | 6 | N/A | 1 | M | GSE60408 |
| OCI-Ly7 | B-cell lymphoma | 12 | N/A | 1 | M | GSE60408 |
| OCI-Ly7 | B-cell lymphoma | 24 | N/A | 1 | M | GSE60408 |
| OHT-resistent MCF-7 | Breast | 4 | 10nM | 4 | F | GSE5840 |
| Panc-1ER | Pancreas | 3 | 10nM | 1 | M | GSE28901 |
| Panc-1ER | Pancreas | 24 | 10nM | 1 | M | GSE28902 |
| Panc-1GLI1ER | Pancreas | 3 | 10nM | 1 | M | GSE28901 |
| Panc-1GLI1ER | Pancreas | 24 | 10nM | 1 | M | GSE28902 |
| PC3ER | Prostate | 24 | 10nM | 1 | M | GSE28899 |
| PC3ER | Prostate | 24 | 10nM | 1 | M | GSE28900 |
| PC3GLI1ER | Prostate | 24 | 10nM | 1 | M | GSE28899 |
| PC3GLI1ER | Prostate | 24 | 10nM | 1 | M | GSE28900 |
| PEO1 | Ovary | 3 | 1nM | 4 | F | GSE81612 |
| PEO4 | Ovary | 3 | 1nM | 4 | F | GSE81612 |
| Postmenupausal Endometrium | Endometrium | 84 | 2mg p.o. | 7 | F | GSE12446 |
| Primary Atrial Cells | Heart | 24 | 10nM | 6 | Mixture | E-MEXP-2971 |
| Primary neuronal cells | Neuron | 168 | 2uM | 1 | N/A | GSE17645 |
| Primary Osteoblasts | Bone | 24 | 10nM | 3 | N/A | E-MEXP-1053 |
| Primary uterine gland organoid | Uterus | 48 | 10 nM | 3 | F | GSE136795 |
| Prostaspheres | Prostate | 168 | 100pM | 3 | M | GSE62953 |
| Random periareolar fine needle aspirates | Breast | 168 | N/A | 8 | F | GSE10270 |
| siDOT1L MCF-7 | Breast | N/A | 10nM | 3 | F | E-MTAB-6893 |
| SKBR3 | Breast | 1 | 1uM | 3 | F | GSE11567 |
| SKBR3 | Breast | 3 | 1uM | 1 | F | GSE32669 |
| SKBR3 | Breast | 3 | 1uM | 1 | F | GSE39719 |
| SKOV3 | Ovary | 24 | 20 nM | 2 | F | GSE115481 |
| SKOV3 | Ovary | 24 | 20 nM | 2 | F | GSE115481 |
| SUM149PT | Breast | 48 | 10nM | 3 | F | GSE28542 |
| SUM190PT | Breast | 48 | 10nM | 3 | F | GSE28542 |
| SUM44 | Breast | 3 | 1nM | 4 | F | GSE50694 |
| SUM44 | Breast | 24 | 1nM | 4 | F | GSE50694 |
| SUM44 | Breast | N/A | 1nM | 3 | F | GSE100075 |
| SUM44 | Breast | N/A | 1nM | 3 | F | GSE75971 |
| SUM52PE | Breast | 6 | 1nM | 3 | F | GSE70759 |
| SW480 | Colon | N/A | 10nM | 4 | M | E-MEXP-1473 |
| T47-D | Breast | 4 | 10nM | 3 | F | GSE108304 |
| T47-D | Breast | 24 | 10nM | 2 | F | GSE3529 |
| T47-D | Breast | 24 | 10nM | 3 | F | GSE108304 |
| T-47D | Breast | 1 | 10nM | 2 | F | GSE7206 |
| T-47D | Breast | 1 | N/A | 2 | F | GSE3834 |
| T-47D | Breast | 2 | N/A | 2 | F | GSE3834 |
| T-47D | Breast | 3 | 100pM | 2 | F | GSE89700 |
| T-47D | Breast | 3 | 100pM | 2 | F | GSE89700 |
| T-47D | Breast | 3 | 1uM | 1 | F | GSE32666 |
| T-47D | Breast | 3 | 1uM | 1 | F | GSE39720 |
| T-47D | Breast | 3 | 1uM | 1 | F | GSE60878 |
| T-47D | Breast | 3 | N/A | 12 | F | GSE107863 |
| T-47D | Breast | 4 | 10nM | 2 | F | GSE93443 |
| T-47D | Breast | 4 | N/A | 2 | F | GSE3834 |
| T-47D | Breast | 6 | 10nM | 2 | F | GSE7206 |
| T-47D | Breast | 6 | 10nM | 3 | F | GSE74032 |
| T-47D | Breast | 6 | 1nM | 3 | F | GSE70759 |
| T-47D | Breast | 6 | N/A | 10 | F | GSE107863 |
| T-47D | Breast | 8 | 10nM | 2 | F | GSE38234 |
| T-47D | Breast | 8 | N/A | 2 | F | GSE3834 |
| T-47D | Breast | 12 | 10nM | 1 | F | GSE80358 |
| T-47D | Breast | 12 | 10nM | 1 | F | GSE80366 |
| T-47D | Breast | 12 | 10nM | 2 | F | GSE7206 |
| T-47D | Breast | 12 | N/A | 2 | F | GSE3834 |
| T-47D | Breast | 12 | N/A | 10 | F | GSE107863 |
| T-47D | Breast | 16 | 10 nM | 1 | F | GSE127760 |
| T-47D | Breast | 16 | 10 nM | 1 | F | GSE127760 |
| T-47D | Breast | 16 | 10 nM | 1 | F | GSE127760 |
| T-47D | Breast | 16 | 10 nM | 1 | F | GSE127760 |
| T-47D | Breast | 16 | 10nM | 2 | F | GSE7206 |
| T-47D | Breast | 16 | 10nM | 3 | F | GSE62243 |
| T-47D | Breast | 24 | 1 nM | 2 | F | GSE118942 |
| T-47D | Breast | 24 | 1 nM | 2 | F | GSE118942 |
| T-47D | Breast | 24 | 10nM | 2 | F | GSE45047 |
| T-47D | Breast | 24 | 10nM | 3 | F | E-MTAB-4975 |
| T-47D | Breast | 24 | 10nM | 3 | F | GSE99626 |
| T-47D | Breast | 24 | 1nM | 4 | F | GSE89888 |
| T-47D | Breast | 24 | 1nM | 6 | F | GSE69774 |
| T-47D | Breast | 24 | N/A | 2 | F | GSE3834 |
| T-47D | Breast | 30 | 10nM | 2 | F | GSE7206 |
| T-47D | Breast | 48 | 1nM | 2 | F | GSE77345 |
| T-47D | Breast | N/A | 1nM | 3 | F | GSE75971 |
| T-47D | Breast | N/A | N/A | 2 | F | GSE3529 |
| T-47D pLB | Breast | 72 | 1nM | 3 | F | GSE55350 |
| T-47D pLUM | Breast | 72 | 1nM | 3 | F | GSE55350 |
| T-47D Y537S | Breast | N/A | N/A | 3 | F | GSE94493 |
| T47D/A18 | Breast | 4032 | 1nM | 2 | F | GSE85536 |
| T47D/A18 | Breast | 6384 | 1nM | 2 | F | GSE85536 |
| T47D-MTVL | Breast | 6 | 10nM | 1 | F | GSE53463 |
| Tamoxifen-resistent MCF-7 | Breast | 1 | 10nM | 1 | F | GSE21618 |
| Tamoxifen-resistent MCF-7 | Breast | 2 | 10nM | 1 | F | GSE21618 |
| Tamoxifen-resistent MCF-7 | Breast | 3 | 10nM | 1 | F | GSE21618 |
| Tamoxifen-resistent MCF-7 | Breast | 6 | 10nM | 1 | F | GSE21618 |
| Tamoxifen-resistent MCF-7 | Breast | 12 | 10nM | 1 | F | GSE21618 |
| Tamoxifen-resistent MCF-7 | Breast | 24 | 10nM | 7 | F | GSE21618 |
| Tamoxifen-resistent MCF-7 | Breast | 48 | 10nM | 1 | F | GSE21618 |
| TDG | Breast | 4 | 10nM | 3 | F | GSE108304 |
| TDG | Breast | 24 | 10nM | 3 | F | GSE108304 |
| Term Decidual Cells | Decidua | 6 | 10nM | 3 | F | GSE65835 |
| TYS | Breast | 4 | 10nM | 3 | F | GSE108304 |
| TYS | Breast | 24 | 10nM | 3 | F | GSE108304 |
| U2932 | B-cell lymphoma | 6 | N/A | 1 | F | GSE60408 |
| U2932 | B-cell lymphoma | 12 | N/A | 1 | F | GSE60408 |
| U2OS | Osteosarcoma | 4 | 10nM | 2 | F | GSE1153 |
| U2OS | Osteosarcoma | 8 | 10nM | 2 | F | GSE1153 |
| U2OS | Osteosarcoma | 24 | 10nM | 4 | F | GSE2292 |
| U2OS | Bone | 24 | 10nM | 4 | F | GSE2292 |
| U2OS | Osteosarcoma | 48 | 10nM | 2 | F | GSE1153 |
| U2OS-ERa with NCOA1 siRNA | Osteosarcoma | 3 | 10nM | 3 | F | GSE90548 |
| Umbilical cord blood cells | Stem cell | 1 | 10nM | 1 | N/A | GSE4609 |
| Umbilical endothelial cells | Umbilical cord | 120 | 1nM | 5 | F | E-MEXP-1227 |
| Vaginal biopsy | Vagina | 2160 | 0.5 mg/day | 19 | F | GSE11622 |
| ZR75 | Breast | 12 | 10nM | 1 | F | GSE80366 |
| ZR75 | Breast | N/A | 1nM | 3 | F | GSE75971 |
| ZR75.1 | Breast | 1 | 10nM | 2 | F | E-TABM-631 |
| ZR75.1 | Breast | 1 | 10nM | 2 | F | E-TABM-742 |
| ZR75.1 | Breast | 2 | 10nM | 2 | F | E-TABM-631 |
| ZR75.1 | Breast | 2 | 10nM | 2 | F | E-TABM-742 |
| ZR75.1 | Breast | 4 | 10nM | 2 | F | E-TABM-742 |
| ZR75.1 | Breast | 4 | 10nM | 3 | F | E-TABM-631 |
| ZR75.1 | Breast | 6 | 10nM | 2 | F | E-TABM-1194 |
| ZR75.1 | Breast | 6 | 10nM | 2 | F | E-TABM-631 |
| ZR75.1 | Breast | 6 | 10nM | 2 | F | E-TABM-742 |
| ZR75.1 | Breast | 8 | 10nM | 2 | F | E-TABM-631 |
| ZR75.1 | Breast | 8 | 10nM | 2 | F | E-TABM-742 |
| ZR75.1 | Breast | 12 | 10nM | 2 | F | E-TABM-1194 |
| ZR75.1 | Breast | 12 | 10nM | 2 | F | E-TABM-631 |
| ZR75.1 | Breast | 12 | 10nM | 2 | F | E-TABM-742 |
| ZR75.1 | Breast | 16 | 10nM | 2 | F | E-TABM-631 |
| ZR75.1 | Breast | 16 | 10nM | 2 | F | E-TABM-742 |
| ZR75.1 | Breast | 20 | 10nM | 2 | F | E-TABM-631 |
| ZR75.1 | Breast | 20 | 10nM | 2 | F | E-TABM-742 |
| ZR75.1 | Breast | 24 | 10nM | 2 | F | E-TABM-1194 |
| ZR75.1 | Breast | 24 | 10nM | 2 | F | E-TABM-631 |
| ZR75.1 | Breast | 24 | 10nM | 2 | F | E-TABM-742 |
| ZR75.1 | Breast | 28 | 10nM | 2 | F | E-TABM-631 |
| ZR75.1 | Breast | 28 | 10nM | 2 | F | E-TABM-742 |
| ZR75.1 | Breast | 32 | 10nM | 2 | F | E-TABM-631 |
| ZR75.1 | Breast | 32 | 10nM | 2 | F | E-TABM-742 |
| ZR75.1 | Breast | 72 | 10nM | 2 | F | E-TABM-1194 |
| ZR-75.1 | Breast | 1 | 50nM | 2 | F | GSE1864 |
| ZR-75.1 | Breast | 1 | 50nM | 2 | F | GSE1864 |
| ZR-75.1 | Breast | 2 | 50nM | 2 | F | GSE1864 |
| ZR-75.1 | Breast | 2 | 50nM | 3 | F | GSE1864 |
| ZR-75.1 | Breast | 4 | 50nM | 2 | F | GSE1864 |
| ZR-75.1 | Breast | 4 | 50nM | 2 | F | GSE1864 |
| ZR-75.1 | Breast | 6 | 50nM | 2 | F | GSE1864 |
| ZR-75.1 | Breast | 6 | 50nM | 2 | F | GSE1864 |
| ZR-75.1 | Breast | 8 | 50nM | 2 | F | GSE1864 |
| ZR-75.1 | Breast | 8 | 50nM | 3 | F | GSE1864 |
| ZR-75.1 | Breast | 12 | 50nM | 2 | F | GSE1864 |
| ZR-75.1 | Breast | 12 | 50nM | 2 | F | GSE1864 |
| ZR-75.1 | Breast | 16 | 50nM | 2 | F | GSE1864 |
| ZR-75.1 | Breast | 16 | 50nM | 3 | F | GSE1864 |
| ZR-75.1 | Breast | 20 | 50nM | 2 | F | GSE1864 |
| ZR-75.1 | Breast | 20 | 50nM | 2 | F | GSE1864 |
| ZR-75.1 | Breast | 24 | 50nM | 2 | F | GSE1864 |
| ZR-75.1 | Breast | 24 | 50nM | 2 | F | GSE1864 |
| ZR-75.1 | Breast | 28 | 50nM | 2 | F | GSE1864 |
| ZR-75.1 | Breast | 28 | 50nM | 2 | F | GSE1864 |
| ZR-75.1 | Breast | 32 | 50nM | 2 | F | GSE1864 |
| ZR-75.1 | Breast | 32 | 50nM | 2 | F | GSE1864 |
| ZR-75-1 | Breast | 16 | 10nM | 4 | F | GSE38132 |
| ZR-75-1 | Breast | 16 | 10nM | 4 | F | GSE61368 |

Supplemental Table 5. The number of uniquely regulated genes per xenoestrogen in HepG2 and HepRG cells.

| HepG2 Cells | | | |
| --- | --- | --- | --- |
| Compound | Total Regulated Genes | Unique Regulated Genes | % Unique Genes |
| Estradiol | 2518 | 1085 | 43% |
| EE2 | 4453 | 1068 | 24% |
| TCDD | 4544 | 1527 | 34% |
| DES | 983 | 447 | 45% |
| BPA | 5374 | 1545 | 29% |
| GEN | 3800 | 1076 | 28% |

| HepRG Cells | | | |
| --- | --- | --- | --- |
| Compound | Total Regulated Genes | Unique Regulated Genes | % Unique Genes |
| Estradiol | 2736 | 866 | 32% |
| EE2 | 6581 | 842 | 13% |
| TCDD | 5792 | 2078 | 36% |
| BPA | 6491 | 791 | 12% |
| GEN | 6986 | 1013 | 14% |
