# Supplementary material for: Gene X environment: the cellular environment governs the transcriptional response to environmental chemicals
Source: Hum Genomics. 2020 May 24;14:19. doi: 10.1186/s40246-020-00269-1 (PMC7247264; doi:10.1186/s40246-020-00269-1)
Supplement: Supplementary file 2 — Additional file 2: Supplemental Figures [file 40246_2020_269_MOESM2_ESM.pptx]

## Slide 1
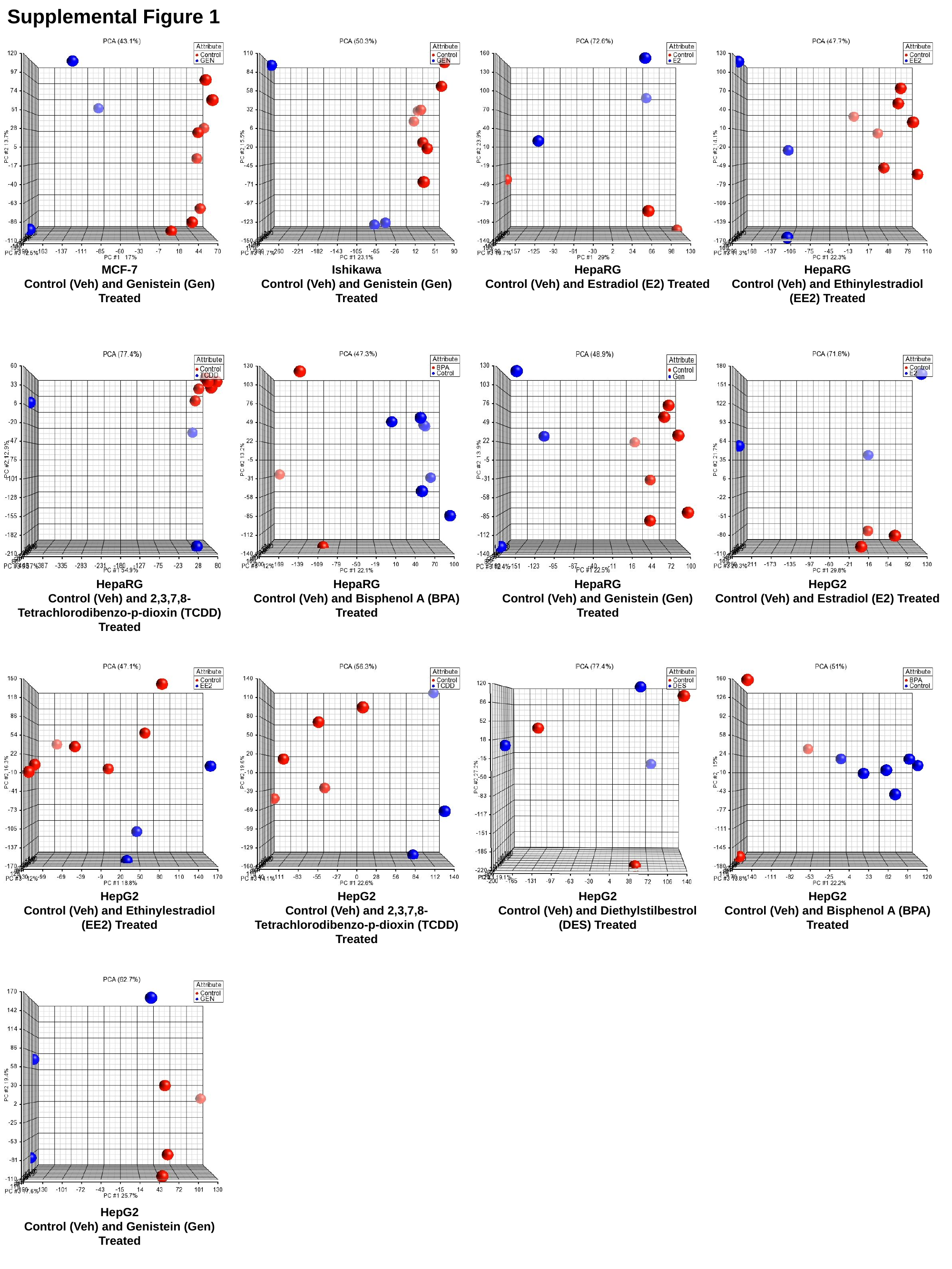

Supplemental Figure 1
MCF-7
Control (Veh) and Genistein (Gen) Treated
Ishikawa
Control (Veh) and Genistein (Gen) Treated
HepaRG
Control (Veh) and Estradiol (E2) Treated
HepaRG
Control (Veh) and Ethinylestradiol (EE2) Treated
HepaRG
Control (Veh) and 2,3,7,8-Tetrachlorodibenzo-p-dioxin (TCDD) Treated
HepaRG
Control (Veh) and Bisphenol A (BPA) Treated
HepaRG
Control (Veh) and Genistein (Gen) Treated
HepG2
Control (Veh) and Estradiol (E2) Treated
HepG2
Control (Veh) and Ethinylestradiol (EE2) Treated
HepG2
Control (Veh) and 2,3,7,8-Tetrachlorodibenzo-p-dioxin (TCDD) Treated
HepG2
Control (Veh) and Diethylstilbestrol (DES) Treated
HepG2
Control (Veh) and Bisphenol A (BPA) Treated
HepG2
Control (Veh) and Genistein (Gen) Treated

## Slide 2
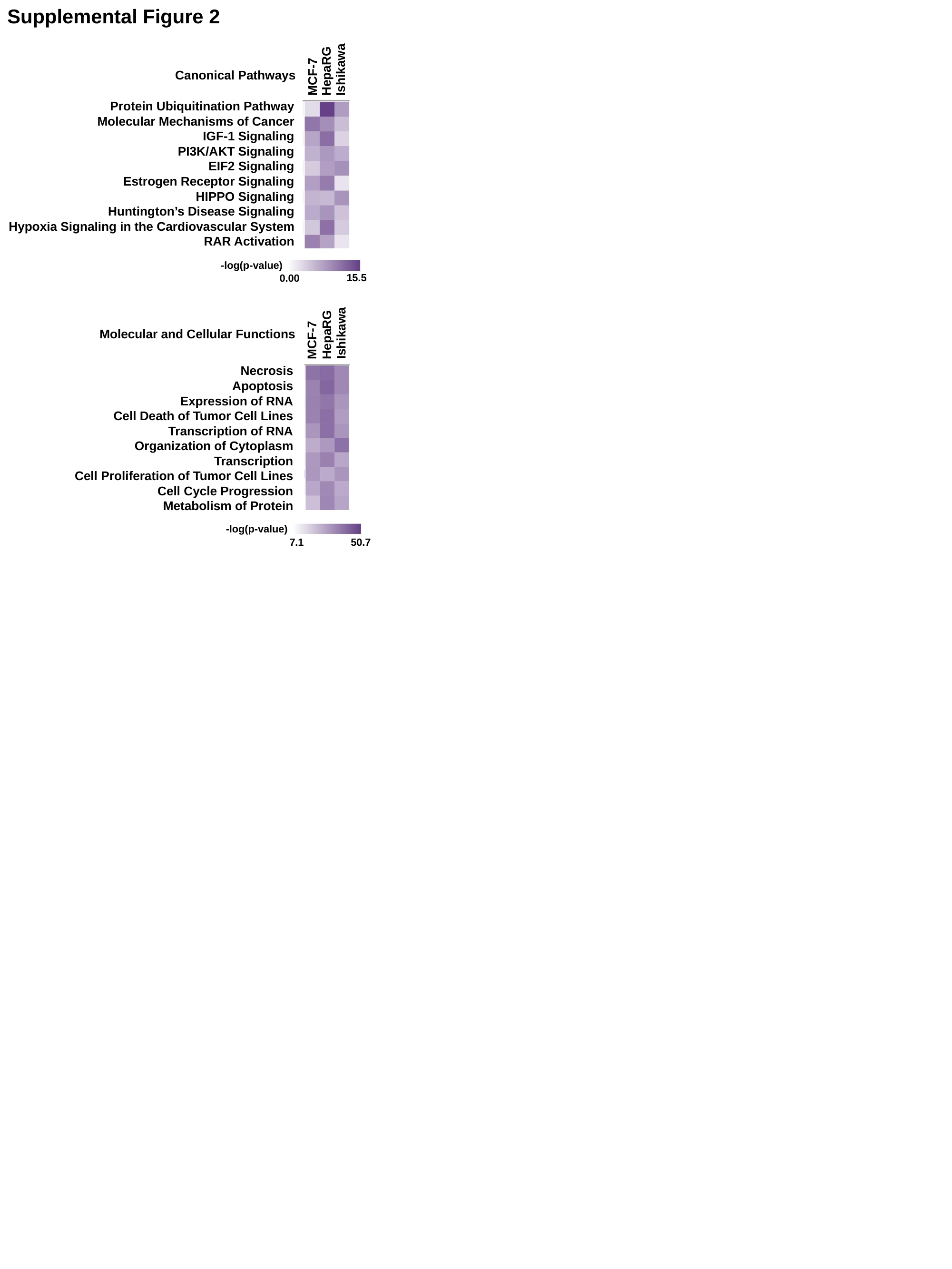

v
Supplemental Figure 2
Ishikawa
HepaRG
Canonical Pathways
MCF-7
Protein Ubiquitination Pathway
Molecular Mechanisms of Cancer
IGF-1 Signaling
PI3K/AKT Signaling
EIF2 Signaling
Estrogen Receptor Signaling
HIPPO Signaling
Huntington’s Disease Signaling
Hypoxia Signaling in the Cardiovascular System
RAR Activation
-log(p-value)
15.5
0.00
Ishikawa
Molecular and Cellular Functions
HepaRG
MCF-7
Necrosis
Apoptosis
Expression of RNA
Cell Death of Tumor Cell Lines
Transcription of RNA
Organization of Cytoplasm
Transcription
Cell Proliferation of Tumor Cell Lines
Cell Cycle Progression
Metabolism of Protein
-log(p-value)
7.1
50.7

## Slide 3
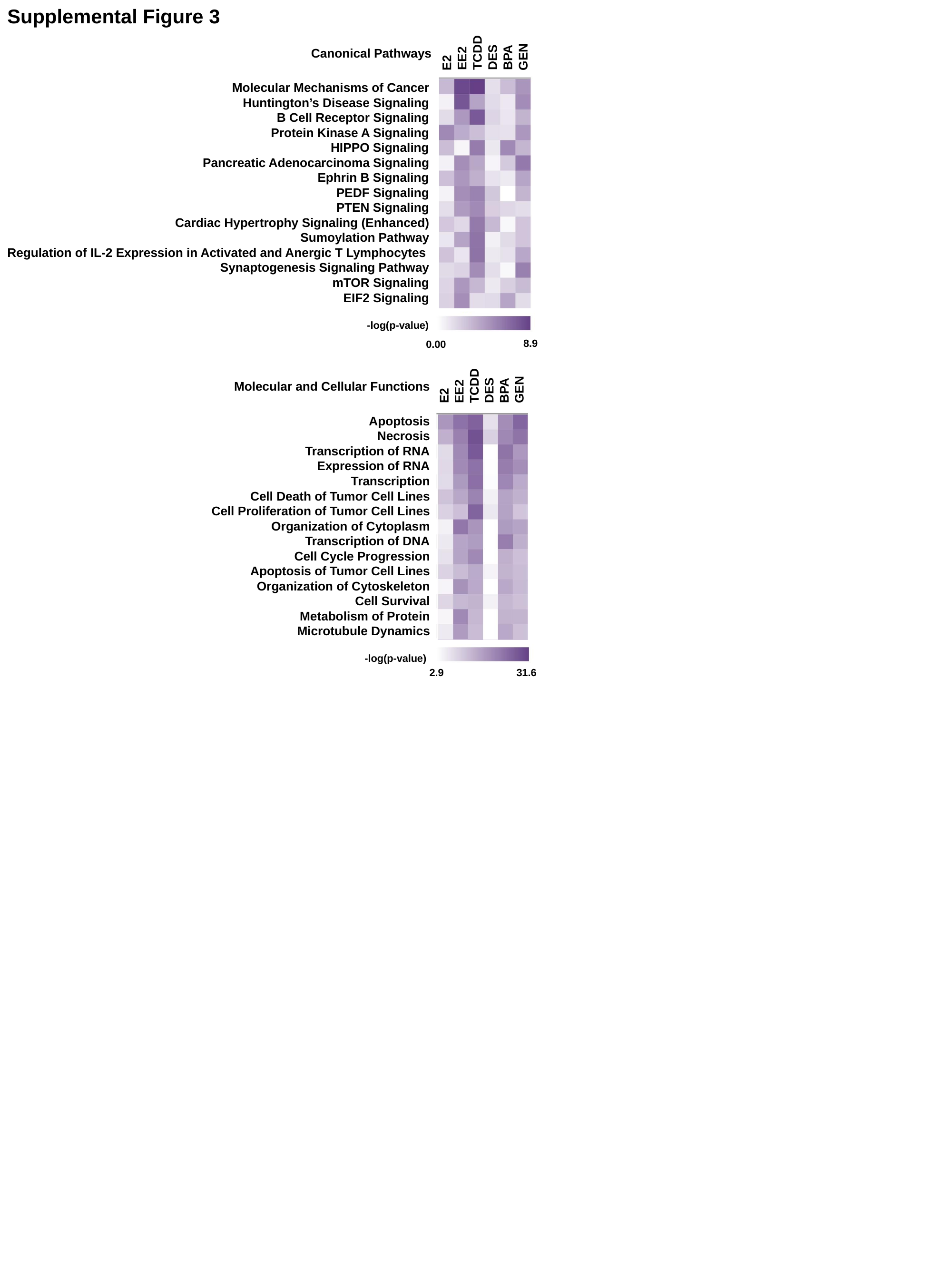

Supplemental Figure 3
TCDD
Canonical Pathways
GEN
DES
BPA
EE2
E2
Molecular Mechanisms of Cancer
Huntington’s Disease Signaling
B Cell Receptor Signaling
Protein Kinase A Signaling
HIPPO Signaling
Pancreatic Adenocarcinoma Signaling
Ephrin B Signaling
PEDF Signaling
PTEN Signaling
Cardiac Hypertrophy Signaling (Enhanced)
Sumoylation Pathway
Regulation of IL-2 Expression in Activated and Anergic T Lymphocytes
Synaptogenesis Signaling Pathway
mTOR Signaling
EIF2 Signaling
-log(p-value)
8.9
0.00
TCDD
Molecular and Cellular Functions
GEN
DES
BPA
EE2
E2
Apoptosis
Necrosis
Transcription of RNA
Expression of RNA
Transcription
Cell Death of Tumor Cell Lines
Cell Proliferation of Tumor Cell Lines
Organization of Cytoplasm
Transcription of DNA
Cell Cycle Progression
Apoptosis of Tumor Cell Lines
Organization of Cytoskeleton
Cell Survival
Metabolism of Protein
Microtubule Dynamics
-log(p-value)
2.9
31.6

## Slide 4
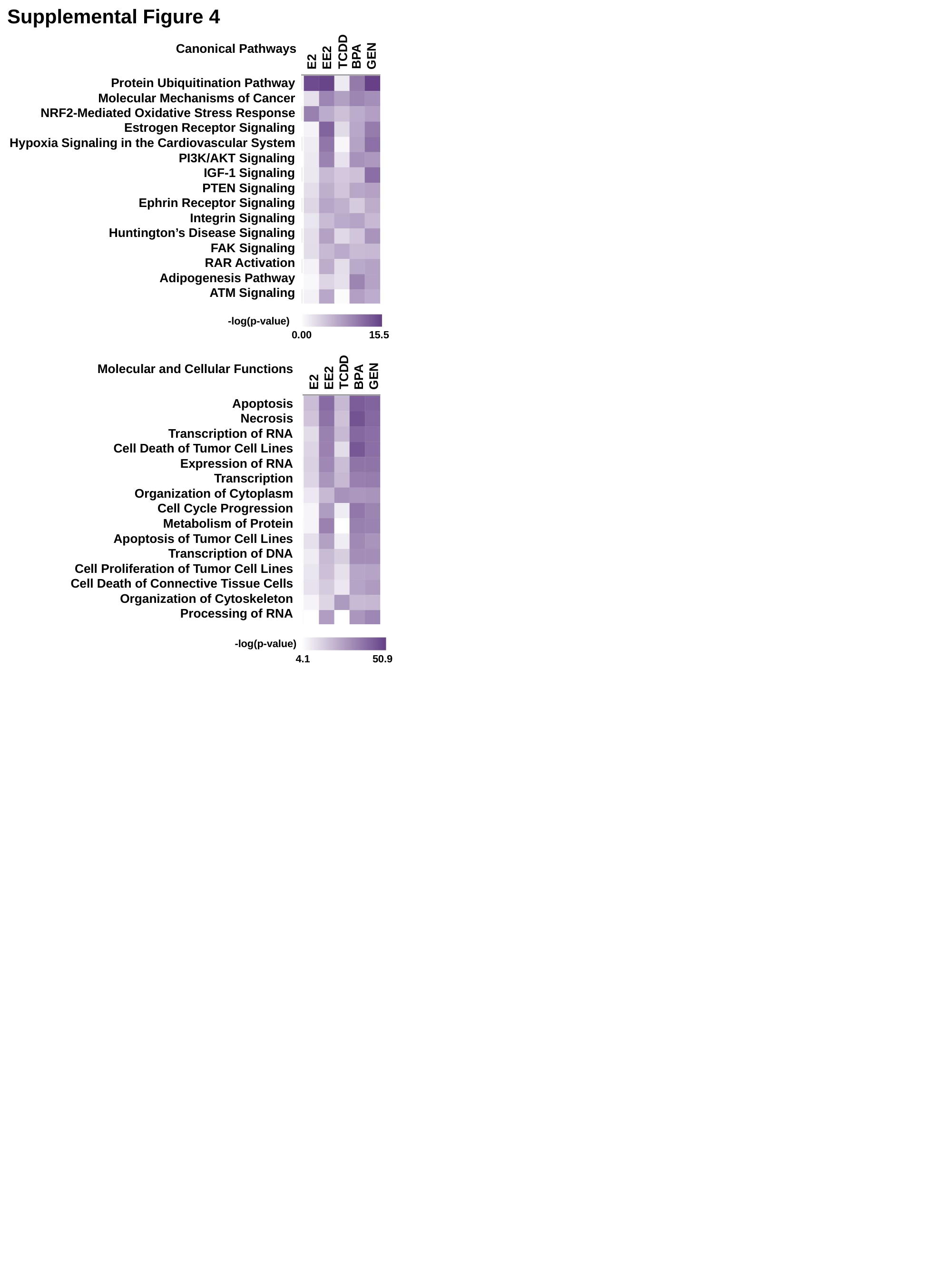

Supplemental Figure 4
Canonical Pathways
TCDD
GEN
BPA
EE2
E2
Protein Ubiquitination Pathway
Molecular Mechanisms of Cancer
NRF2-Mediated Oxidative Stress Response
Estrogen Receptor Signaling
Hypoxia Signaling in the Cardiovascular System
PI3K/AKT Signaling
IGF-1 Signaling
PTEN Signaling
Ephrin Receptor Signaling
Integrin Signaling
Huntington’s Disease Signaling
FAK Signaling
RAR Activation
Adipogenesis Pathway
ATM Signaling
-log(p-value)
0.00
15.5
Molecular and Cellular Functions
TCDD
GEN
BPA
EE2
E2
Apoptosis
Necrosis
Transcription of RNA
Cell Death of Tumor Cell Lines
Expression of RNA
Transcription
Organization of Cytoplasm
Cell Cycle Progression
Metabolism of Protein
Apoptosis of Tumor Cell Lines
Transcription of DNA
Cell Proliferation of Tumor Cell Lines
Cell Death of Connective Tissue Cells
Organization of Cytoskeleton
Processing of RNA
-log(p-value)
50.9
4.1

## Slide 5
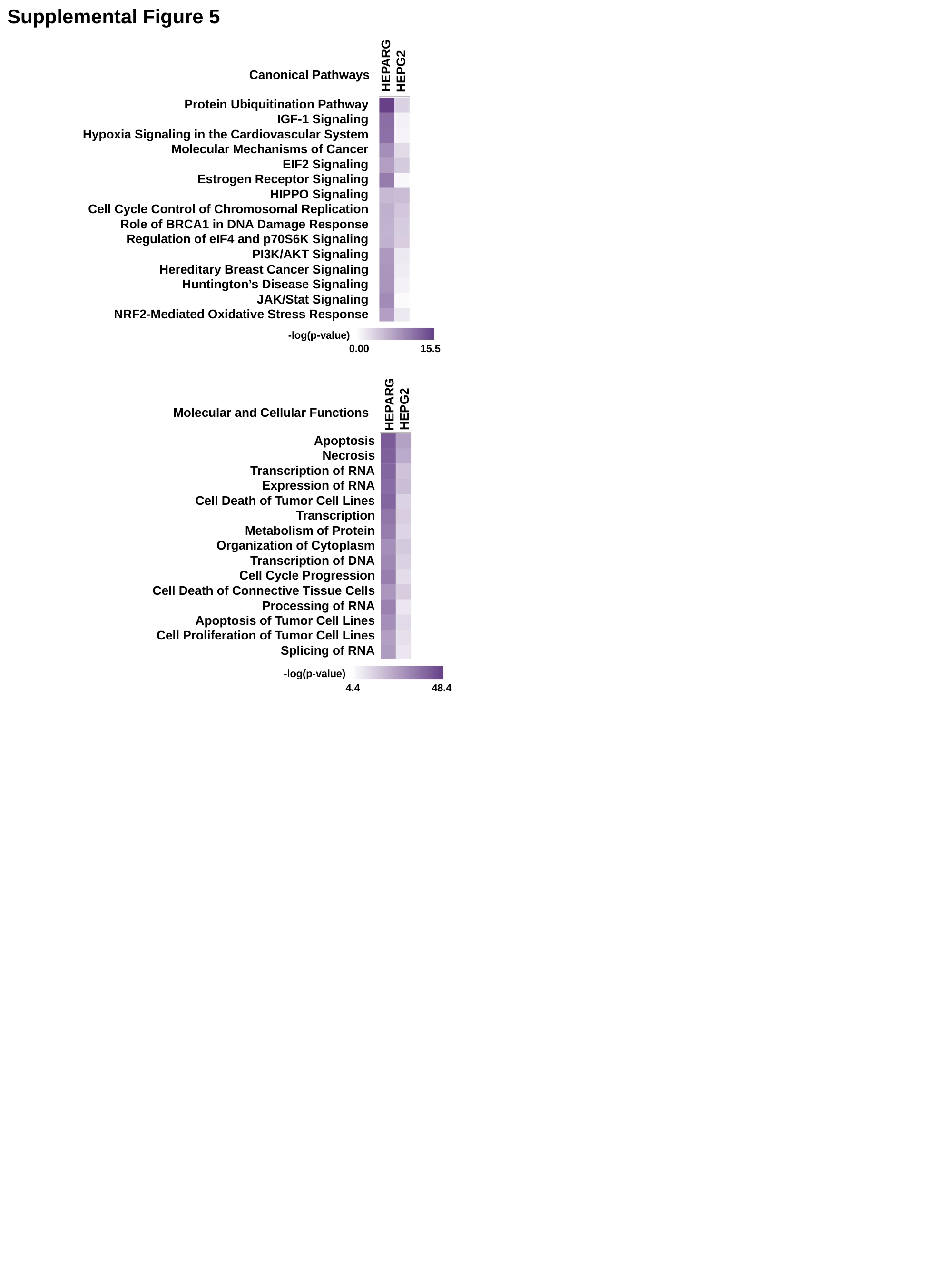

Supplemental Figure 5
HEPARG
HEPG2
Canonical Pathways
Protein Ubiquitination Pathway
IGF-1 Signaling
Hypoxia Signaling in the Cardiovascular System
Molecular Mechanisms of Cancer
EIF2 Signaling
Estrogen Receptor Signaling
HIPPO Signaling
Cell Cycle Control of Chromosomal Replication
Role of BRCA1 in DNA Damage Response
Regulation of eIF4 and p70S6K Signaling
PI3K/AKT Signaling
Hereditary Breast Cancer Signaling
Huntington’s Disease Signaling
JAK/Stat Signaling
NRF2-Mediated Oxidative Stress Response
-log(p-value)
0.00
15.5
HEPARG
HEPG2
Molecular and Cellular Functions
Apoptosis
Necrosis
Transcription of RNA
Expression of RNA
Cell Death of Tumor Cell Lines
Transcription
Metabolism of Protein
Organization of Cytoplasm
Transcription of DNA
Cell Cycle Progression
Cell Death of Connective Tissue Cells
Processing of RNA
Apoptosis of Tumor Cell Lines
Cell Proliferation of Tumor Cell Lines
Splicing of RNA
-log(p-value)
48.4
4.4
